# High-Resolution Sequence-Function Mapping of Full-Length Proteins

**DOI:** 10.1371/journal.pone.0118193

**Published:** 2015-03-19

**Authors:** Caitlin A. Kowalsky, Justin R. Klesmith, James A. Stapleton, Vince Kelly, Nolan Reichkitzer, Timothy A. Whitehead

**Affiliations:** 1 Department of Chemical Engineering and Materials Science, Michigan State University, East Lansing, Michigan, United States of America; 2 Department of Biochemistry and Molecular Biology, Michigan State University, East Lansing, Michigan, United States of America; 3 Department of Biosystems and Agricultural Engineering, Michigan State University, East Lansing, Michigan, United States of America; Weizmann Institute of Science, ISRAEL

## Abstract

Comprehensive sequence-function mapping involves detailing the fitness contribution of every possible single mutation to a gene by comparing the abundance of each library variant before and after selection for the phenotype of interest. Deep sequencing of library DNA allows frequency reconstruction for tens of thousands of variants in a single experiment, yet short read lengths of current sequencers makes it challenging to probe genes encoding full-length proteins. Here we extend the scope of sequence-function maps to entire protein sequences with a modular, universal sequence tiling method. We demonstrate the approach with both growth-based selections and FACS screening, offer parameters and best practices that simplify design of experiments, and present analytical solutions to normalize data across independent selections. Using this protocol, sequence-function maps covering full sequences can be obtained in four to six weeks. Best practices introduced in this manuscript are fully compatible with, and complementary to, other recently published sequence-function mapping protocols.

## Introduction

The amino acid sequence of a protein defines its function, yet our understanding of the contribution of each amino acid to overall activity remains incomplete. As a result, current computational and experimental methods of designing functional proteins have success rates significantly less than 10% [1]. Random directed evolution approaches provide activity improvements, but require high throughputs because about 98% of amino acid substitutions are either deleterious or neutral with respect to the desired function or specific fold [2]. Traditional methods for probing sequence-function relationships, such as alanine scanning and site-saturation mutagenesis, are laborious and inefficient [3–6]. A systematic method to survey the sequence-function space of large proteins would facilitate enzymatic efficiency improvements, antibody-epitope mapping, rapid antibody-antigen maturation, and fine-tuning of computationally designed proteins [7–10].

About a decade ago, Pal et al. introduced a quantitative scanning method to map the energetic landscapes of protein-protein interactions [11]. Libraries were created using saturation mutagenesis at multiple positions and screened with phage display to enrich the population in mutants with enhanced function at the expense of those with impaired function. The complete library was sequenced before and after selection, and comparisons of these frequencies gave a measure of activity for each variant. More recently, Fowler et al. used a similar framework to develop deep mutational scanning [12,13]. In a key step, deep sequencing is used to quantify the frequency of each mutant in the library before and after selection, and the resulting enrichment ratio provides a fitness metric. The ability to sequence millions of sequences in a library allows quantification of thousands of protein variants in a single experiment. Independently, Hietpas et al. developed a similar technique termed EMPIRIC, which they applied to measure fitness effects of point mutations of regions of genes in yeast [14,15]. Since the introduction of deep mutational scanning, similar methods have been applied to characterize protein-ligand interactions and chaperone protein function [11,16]. In a recent report demonstrating the power of the approach, Firnberg et al. produced a comprehensive map of nearly all possible single mutations to a full-length protein, TEM-1β-Lactamase [17]. By combining comprehensive single-site mutagenesis with selection through antibiotic resistance they were able to assess the fitness of 5,760 different mutant protein sequences in a single experiment.

Deep mutational scanning methods were extended to protein engineering applications by Whitehead et al., who applied the deep mutational scanning technique to enhance the affinity and specificity of two designed influenza inhibitors [9]. Deep mutational scanning has since been applied in many different areas of protein engineering including specificity switches and protein stability [18,19].

Given the demonstrated utility and growing popularity of deep mutational scanning as a tool to understand and optimize protein function, we sought to develop a standardized protocol for resolving the sequence determinants of function for full-length proteins. In this contribution, we develop and validate experimental methods for mutant library creation, functional selections, and sequencing library preparation. We derive equations that allow direct, quantitative comparisons across different populations in growth-based selections and fluorescence activated cell sorting (FACS), enabling optimal selection criteria to be determined for these versatile selection techniques. We introduce a gene tiling technique which splits a long gene sequence into several independent libraries, each of which contain a mutated region short enough to be covered with a paired-end read [20]. This approach, combined with the equations developed herein, allow for the unambiguous reconstruction of the sequence-function determinants of full-length proteins. Key considerations for each step in the process are discussed.

## Materials and Methods

### Constructs

**Strains**. E. coli strains used in this study: **XL1-Blue** (Agilent, Santa Clara, CA) recA1 endA1 gyrA96 thi-1 hsdR17 supE44 relA1 lac [F’ proAB lacI1^q^ZΔM15 Tn10 (Tet^r^)]; **Tuner** (Novagen, Billerica, MA) F- ompT hsdS_B_ (r_B_- m_B_-) gal dcm lacY1; **K12 CJ236** (NEB) FΔ(HindIII)::cat (Tra+ Pil+ CamR)/ ung-1 relA1 thi-1 spoT1 mcrA.

**Plasmids**. The plasmid pJK_proJK1_LGK was created by inserting a codon-optimized gene encoding levoglucosan kinase (LGK) (Genscript, Piscataway, NJ) with LEHHHHHH as 95 the C-terminal tag into a pJK-series plasmid [20] using flanking NdeI/XhoI restriction sites. The plasmid pJK_proJK1_kanR_LGK was created by switching the ampR with a kanR resistance cassette using Gibson cloning [21]. Full sequences of both plasmids are given in S1 Note. pJK_eGFP-series plasmids are from a previous study and are listed in S1 Table [22].

### Pfunkel Mutagenesis

Single-site saturation mutagenesis primers containing an NNN degenerate codon were designed in one of two ways: (1.) the online QuikChange Primer Design module (Agilent, Santa Clara, CA); or (2.) primer-design software as detailed in Firnberg et al. [23,24]. Mutagenic libraries were generated from a ssDNA template using the Pfunkel method for comprehensive codon mutagenesis [24]. A separate Pfunkel reaction was performed for each tile region. Protocols were performed as published except the reaction cycling conditions were 95°C for 2 min, followed by 15 cycles of 95°C for 30 sec, 55°C for 45 sec, and 68°C for 15 min. Following the nuclease step the reaction was concentrated using the Zymo Clean and Concentrate kit (Zymo Research, Irvine, CA) and eluted in 6 μL of nanopure water. The entire volume was mixed with 40 μL of electrocompetent XL1-Blue cells (Agilent, Santa Clara, CA). Cells were transformed by electroporation at 1200 V in a 1 mm electroporation cuvette (Eppendorf, Hauppage, NY) with an Eppendorf Eporator. Transformed cells were grown overnight at 37°C on LB agar supplemented with appropriate antibiotic on Nalgene BioAssay plates (245mm × 245mm × 25mm, Sigma Aldrich, St. Louis, MO). Library plasmid DNA was recovered by scraping the BioAssay plate with 5 mL LB, centrifuging the solution to recover the cell pellet, and performing a plasmid midiprep (Qiagen, Valencia, CA) on the cell pellet.

Cells were also plated in serial dilutions from 10^–1^ to 10^–6^ to assess transformation efficiency. Transformation efficiencies ranged from 3x10^5^–1x10^6^ cfu/transformation.

### Secondary Transformations

*E*. *coli* Tuner (Novagen, Billercia, MA) was prepared to be electrocompetent by standard means [25]. Plasmids pJK_proJK1_LGK and pJK_proJK1_kanR_LGK were mixed at a mass ratio of 100:1 respectively. 5–40 ng of the mixed plasmid DNA was transformed into 40 μL of culture by electroporation at 1200 V in 0.1 cm cuvettes (Eppendorf, Hauppage, NY). The reaction was split and plated on ampicillin, kanamycin and ampicillin/kanamycin resistant plates in serial dilutions from 10^–1^ to 10^–6^ and grown overnight. The colonies percentage of double transformants was calculated by dividing the number of CFU’s on the dual antibiotic plate by the CFU’s of the kanamycin plate. This procedure was repeated with library plasmid DNA in place of pJK_proJK1_LGK at a 100:1 ratio to determine the percentage of double transformants.

### Growth-based Selections

Library cell stocks containing mixtures of pJK-series eGFP expression plasmids were thawed on ice and washed with M9 minimal media [22]. Cultures were inoculated to an OD_600_ of 0.03 in M9 minimal media supplemented with 4 g/L glucose and carbenicillin (50 μg/mL). Cultures were grown at 37°C and 250 rpm to an OD_600_ of 0.6. Cell growth was monitored every 45 minutes by OD_600_ measured on a Genesys 20 spectrophotometer (Thermo Fisher Scientific, Waltham, MA). Cells were washed with M9 media. The cells were used to re-inoculate 2.5 mL of fresh media to an OD_600_ of 0.03. Cultures were again grown to an OD_600_ of 0.6 (8.6 total population-averaged generations). Following selection cells were stored in 1mL of M9 media and 7% (v/v) DMSO at -80°C until bacterial plasmid DNA was extracted using a Qiagen miniprep kit (Qiagen, Valencia, CA).

### Primer Design

Two sets of primers were used to amplify stretches of DNA for sequencing. The inner set of primers was designed to be complementary to the regions of DNA at the 5’ and 3’ ends of the gene tile of interest. Forward and reverse primers were designed to have melting temperatures around 55°C. Sequences for the outer, universal set of primers were taken from the TruSeq Small RNA Sample Prep Kit. The outer primers attach the Illumina barcodes and adaptors for sequencing and are listed in S2 Table.

### Gene tile amplification

Gene tiles are amplified by two-step PCR. The contiguous region containing mutations is amplified using tile-specific inner primers using Phusion High Fidelity Polymerase (NEB M0530). The three different methods used to amplify the target region are described in S3 Table. 5 μl of the PCR products were run on a 2% agarose gel and visualized with SYBR-GOLD (Invitrogen) to ensure the presence of a single band of the expected size (~250 bp). Agencourt AMPure XP beads (Beckman Coulter, Brea, CA) were used per the manufacturer’s protocol to purify the PCR product. Samples were multiplexed using index sequences on the outer primers.

DNA concentrations were quantified using Quant-iT PicoGreen (Life Technologies, Carlsbad, CA) quantification and samples were mixed in equimolar quantities for sequencing. Library DNA was sequenced on an Illumina MiSeq with 150-bp PE reads.

### Data Analysis

Enrich 0.2 software was used to compute enrichment ratios of individual mutants from the raw Illumina sequencing files [26]. Forward and reverse reads obtained for each section were used as input. Modifications were made to Enrich 0.2 in order to accommodate shifted and shortened protein alignment sequences (S1 Script). Enrichment ratios that were obtained were normalized as detailed below using custom scripts.

## Theory

### Normalization for Growth Rate Selections

When cells grow exponentially, the specific growth rate, *μ*_*i*_, of any individual mutant *i* can be written as:

$$\mu_{i}=\ln\left(\frac{x_{fi}}{x_{oi}}\right)\frac{1}{t}$$ (1)

Where *x*_*fi*_ is the final concentration of the mutant, *x*_*oi*_ is the initial concentration, and *t* is the time difference between the initial and final concentration of cells. In this formulation we are explicitly neglecting the effect of lag phases for growth. The equation for calculating the enrichment ratio, *ε*_*i*_, of the same mutant is:

$$\varepsilon_{i}=\log_{2}\left(\frac{f_{fi}}{f_{oi}}\right)$$ (2)

Where *f*_*fi*_ is the final frequency of the mutant in the library population and *f*_*oi*_ is the initial frequency. These frequencies can be converted to cell concentrations by the equations below:

$$f_{oi}=\frac{x_{oi}}{\sum x_{oi}}$$ (3)

$$f_{fi}=\frac{x_{fi}}{\sum x_{fi}}$$ (4)

Where *Σx*_*oi*_ is the initial concentration of the culture and *Σx*_*fi*_ is the final concentration. The enrichment ratio can be rewritten as:

$$\varepsilon_{i}=\log_{2}\left(\frac{x_{fi}}{x_{oi}}\right)-\log_{2}\left(\frac{\sum x_{fi}}{\sum x_{oi}}\right)$$ (5)

Combining this equation with (1) leads to:

$$\mu_{i}\log_{2}e=\frac{1}{t}\left(\varepsilon_{i}+\log_{2}\left(\frac{\sum x_{fi}}{\sum x_{oi}}\right)\right)$$ (6)

We can define the change in culture density between the initial and final conditions in terms of the number of average doubling periods (*g*_*p*_) according to:

$$\log_{2}\left(\frac{\sum x_{fi}}{\sum x_{oi}}\right)=\#\;of\;Doublings=g_{p}$$ (7)

Similarly, we can remove time from (5) by redefining it as:

$$t=\frac{g_{p}ln2}{\mu_{p}}$$ (8)

where *μ*_*p*_ is equal to the bulk average growth rate of the population between the initial and final conditions.

Combining (7) and (8) into (6) leads to a description of the growth rate of mutant *i* as a function of its enrichment ratio:

$$\mu_{i}={\bar{\mu }}_{p}\left(\frac{\varepsilon_{i}}{g_{p}}+1\right)$$ (9)

It is often helpful to express the fitness of mutant *i*, *ζ*_*i*_, normalized to the growth rate of the starting construct (wild-type; μ_wt_)

$$\zeta_{i}=\log_{2}\left(\frac{\mu_{i}}{\mu_{wt}}\right)$$ (10)

$$\zeta_{i}=\log_{2}\left(\frac{\frac{\varepsilon_{i}}{g_{p}}+1}{\frac{\varepsilon_{wt}}{g_{p}}+1}\right)$$ (11)

Since the starting construct is usually included in the population, fitness of each variant *i* can be normalized across different selection experiments given only the number of doubling periods as well as the enrichment ratios for the mutant and wild-type construct.

We can also rewrite the enrichment ratio as a function of growth rate:

$$\varepsilon_{i}=g_{p}\left(\frac{\mu_{i}}{{\bar{\mu }}_{p}}-1\right)$$ (12)

The enrichment ratio will increase linearly with the number of doubling periods so long as a mutant is able to exceed the population-averaged growth rate.

### Normalization for Fluorescence-Activated Cell Sorting

For comparisons of variants across different populations, we desire a method to reconstruct mean fluorescence for each mutant,${\overset{-}{F}}_{i}$, from its enrichment ratio *ε*_*i*_. In fluorescence-activated cell sorting (FACS), populations are screened by collecting cells with fluorescence above a certain gating threshold. A clonal population of cells will exhibit a mean fluorescence with a certain variance according to cell size, surface density of displayed proteins, or other factors. Thus, only a fraction of cells for each variant will exceed the fluorescence threshold needed for collection. Since fluorescence measurements of clonal population of cells are log-normally distributed in flow cytometry, ${\overset{-}{F}}_{i}$can be determined using regular statistical calculations:

$${\bar{F}}_{i}^{'}=\ln\left(F_{g}\right)-\sigma^{'}\sqrt{2}\;erf^{-1}\left(1-2\frac{x_{fi}}{x_{oi}}\right)$$ (13)

Here, ${\overset{-}{F}}_{i}{\;}^{\prime }$is the mean of the natural log of the fluorescence for variant *i*, *σ´* is the natural log of the standard deviation of the data, *F*_*g*_ is the fluorescence gating threshold for the experiment, and the ratio $\frac{x_{fi}}{x_{oi}}$ is the fraction of variant *i* that is collected above the gating threshold. ${\overset{-}{F}}_{i}{\;}^{\prime }$can be determined from *F*_*wt*_ *´* by:

$${\overset{-}{F}}_{i}=e^{\left({\overset{-}{F}}_{i}^{\prime }+\frac{\sigma^{\prime }{}^{2}}{2}\right)}$$ (14)

It remains to find $\frac{x_{fi}}{x_{oi}}$ in terms of experimentally measurable values. The flow cytometer used to analyze the culture records the percentage of the total population sampled that is collected, *ϕ*. This value can be written as:

$$\phi_{i}=\frac{\sum x_{fi}}{\sum x_{oi}}$$ (15)

From sequencing data, the enrichment ratio of each mutant, ε, is also known and can be written as:

$$2^{\varepsilon_{i}}=\frac{x_{fi}}{x_{oi}}\frac{\sum x_{oi}}{\sum x_{fi}}$$ (16)

Combining Equations (14–16), we end up with:

$$\frac{x_{fi}}{x_{oi}}=\phi 2^{\varepsilon_{i}}$$ (17)

Finally, combining this relation into Equations (13–14) leads to:

$${\bar{F}}_{i}=\exp\left[\frac{\sigma^{'}{}^{2}}{2}+\ln\left(F_{g}\right)-\sigma^{'}\sqrt{2}\;erf^{-1}\left(1-\phi 2^{\varepsilon_{i}+1}\right)\right]$$ (18)

As with the growth-based selection, it is often helpful to express the fitness of mutant *i* normalized to the fluorescence of the starting construct (wild-type;${\overset{-}{F}}_{wt}$)

$$\zeta_{i}=\log_{2}\left(\frac{{\bar{F}}_{i}}{{\bar{F}}_{wt}}\right)$$ (19)

$$\zeta_{i}={\text{log}}_{2}\left(e\right)\sqrt{2}\sigma^{\prime }\left[erf^{-1}\left(1-\phi 2^{\left(\varepsilon_{wt}+1\right)}\right)-erf^{-1}\left(1-\phi 2^{\left(\varepsilon_{i}+1\right)}\right)\right]$$ (20)

To normalize fluorescence measurements, *ϕ* is set by the experiment, and the enrichment ratios *ε*_*i*_ and *ε*_*wt*_ are obtained from analysis of the raw sequencing files. In this derivation we assume that the log-transformed standard deviation is the same between the individual variant and the wild-type sequence. We have not rigorously tested this assumption. Note that the form of the fitness metric used in this work has the standard deviation as a scalar which is unlikely to vary much using the same cell type and flow cytometer; thus, ratios of fluorescence measurements can be related between populations using only the enrichment ratios and the gating threshold.

## Results and Discussion

We have developed a standardized method to map the sequence-function relationships of entire gene sequences encoding full-length proteins. This process is applicable to a wide variety of proteins, including binding proteins, fluorescent proteins, and enzymes. With some modifications, the method can also be extended to membrane proteins and transcription factors. The protein class determines the selection method: binding proteins can be screened or sorted using phage or yeast display techniques, whereas growth-based selections are preferable for enzymes [9,12,17,27,28]. Regardless of the protein category, the initial sequence should encode some level of functional activity as a basis to distinguish active and inactive proteins.

**Fig 1:**
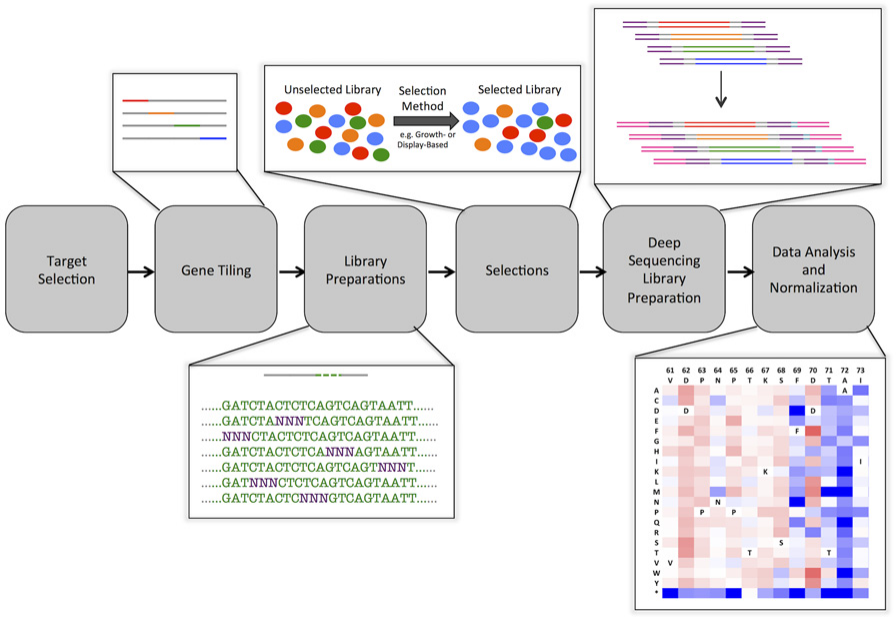
Overview of high-resolution sequence-function mapping process. *Target Selection*. Proteins of interest are selected for interrogation of sequence function relationships. A plasmid containing the gene-encoding sequence is generated. *Gene tiling*. Starting from this gene sequence, semi-overlapping tiles are generated to cover the entire gene. These tiles are either 150, 250 or 300 bp in length in order to be sequenced in paired-end mode on Illumina deep sequencing platforms. *Library Preparation*. The single-pot PFunkel method is used to generate a comprehensive single-site saturation mutagenesis library. *Selections*. Growth-based selections and FACS screens are used to resolve library populations; these selections should not completely converge on a few members of the population. It is important that the initial protein shows activity toward the selection method. *Deep Sequencing and Library Preparation*. After selection, cells are lysed and plasmid DNA is purified. The specific mutated tile region of the gene of interest is then amplified using overhang PCR, at which time Illumina sequencing primers and adaptors with selection-specific indexes are attached. *Data analysis and normalization*. Barcoded DNA is sequenced on a standard Illumina platform, analyzed, and normalized using custom scripts. The end result of this analysis is a comprehensive portrait of the effects of sequence on function for thousands of single point mutants in the gene of interest. These portraits can be used for various purposes such as improving protein binding affinity and specificity or improving enzymatic catalytic efficiency.

Fig. 1 outlines the basic steps covering target selection, gene tiling, library preparation, selection, deep sequencing library preparation, and data analysis and normalization. We have written custom scripts and modified published scripts to facilitate data generation and analysis (S1 and S2 Scripts). Additionally, we have formulated optimal selection criteria and derived equations governing the normalization of results across different selection conditions. Practical considerations for each step are listed in S2 Note. In the following sections we consider each step in the overall process in detail.

### Gene Tiling

**Fig 2:**
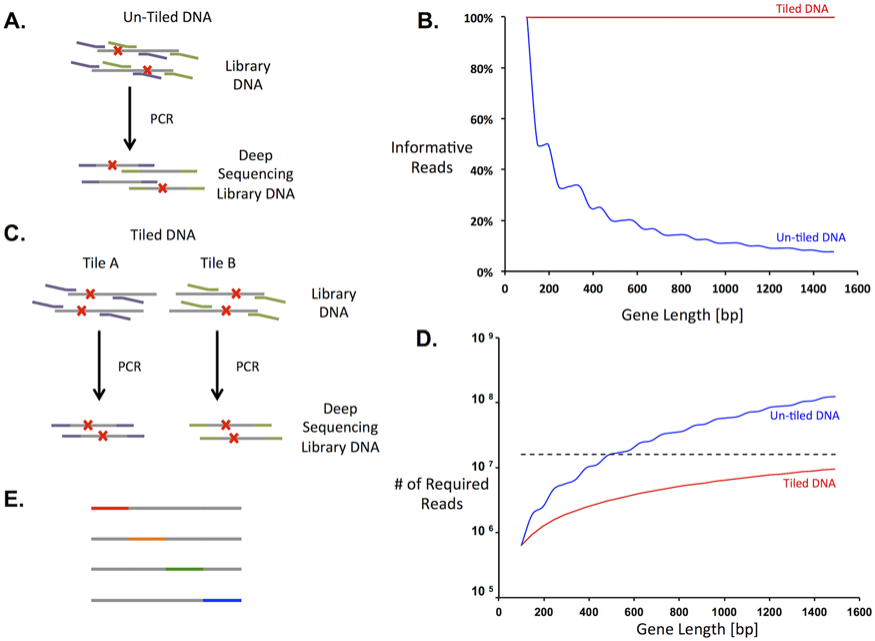
Gene tiling increased the efficiency of deep sequencing for sequence-function mapping. *A*. Deep sequencing without using gene tiles. Gene sequences are represented by grey lines, mutations by red x’s, and sequencing primers by purple and green lines. Several previous methods to amplify target DNA (left) amplify both mutated and non-mutated regions. The latter result in wasted reads and increase the sequencing capacity necessary to resolve the entire library. *B*. Percent of usable reads as a function of gene length with (red line) and without (blue line) gene tiling. *C*. Gene tiling has the ability to reduce the number of DNA sequencing reads necessary by targeting the region with a mutation in PCR amplification for sequencing purposes. To implement gene tiling, separate libraries are prepared and sorted for each tile. *D*. Number of sequence reads required for 300-fold average coverage of nonsynonymous mutations with (red line) or without (blue line) gene tiling. The horizontal dashed line represents the average number of DNA sequences from a single MiSeq lane. *E*. In gene tiling, short contiguous stretches of DNA (tiles) are targeted for mutations. Gene tiles are indicated by the colored dashed lines and cover the entire gene sequence among the different libraries.

A protein of 250 residues is encoded by a gene of 750 bp, which is longer than high-quality read lengths of existing sequencing platforms. Previous approaches to map sequence to function for full-length proteins involved sequencing the entire gene as smaller amplified segments (Fig. 2A) [9]. Because there should be only one mutation per gene, reads from amplified regions other than the one containing the mutation yield no information and are wasted. Fig. 2B shows the percentage of total sequence reads that provide information as a function of gene length. As gene length increases, the percentage of usable sequencing data decreases and, consequently, more reads are needed to ensure proper coverage. For example, using this previous method results in usable information for only 33% of the sequencing reads in a gene of length 450 bp.

We have improved the efficiency of scanning long genes by dividing the gene into multiple “tiles,” each of which is effectively treated as a distinct gene. Each tile is independently mutagenized, subjected to selection, and sequenced before our analysis pipeline normalizes and merges the count data to generate the sequence-function map of the full gene. Tile regions are designed to be slightly shorter than a sequencing read, and within each parallel mutagenesis reaction, mutations are restricted to the corresponding tile. For example, tiles designed for 150-bp read lengths would consist of a central 120-bp mutated region flanked by 15-bp constant regions for PCR primer annealing. Multiple, partially overlapping libraries are prepared for each gene to ensure full coverage of the protein. This approach eliminates excess wild-type sequencing because only the region containing the mutation is sequenced (Fig. 2C). However, the tradeoff is that assessing the function of a full-length sequence requires multiple independent selections. Since population dynamics may vary among selections the enrichment ratios must be normalized to allow comparisons across tiles (see Theory section and below).

### Library Mutagenesis Preparation

Our objective is to map the function of every single nonsynonymous (NS) mutation of a protein-encoding sequence. In an ideal system, 1) There would be exactly one NS mutation per protein-encoding sequence; 2) The library would contain complete uniform coverage of all possible single NS mutations; 3) The library prep method would be as reliable, fast and inexpensive as possible; and 4) Each cell would harbor a single protein-encoding sequence.

Numerous methods have been described for the creation of mutant libraries [29–32]. Certain protocols, like QuikChange or Kunkel mutagenesis, introduce mutations at specified locations with specific primers. Because each residue targeted for mutation requires a separate primer and a separate reaction, creation of a single-site saturation mutagenesis (SSM) library for a 250-residue protein requires 250 unique primers and 250 separate reactions, limiting scalability. A newly developed method named Pfunkel incorporates the benefits of Kunkel mutagenesis while minimizing library preparation time by combining the individual SSM reactions into a single-pot [24].

To evaluate the performance of Pfunkel, we created a SSM library of the first forty residues on a codon-optimized gene encoding levoglucosan kinase (LGK) from *L*. *starkeyi* (GenBank: EU751287.1) [33]. A SSM library incorporating NNN codons should theoretically contain 2520 (63 codons at 40 positions) unique NS mutations. The mutagenesis primer set was manually designed using the Agilent QuikChange primer design calculator, and a Pfunkel reaction was performed essentially as described in Firnberg *et al*. [23,24]. The resulting library was sequenced using 150-bp paired-end (PE) reads on an Illumina MiSeq. The quality of the mutagenesis procedure was evaluated based on the percent coverage of mutations at the DNA and amino-acid levels, the percentage of starting (wild-type) DNA sequences, and the percentage of sequences with more than one mutation in the coding sequence.

Coverage analysis of the SSM library showed 99% of the 2520 possible codon mutations were incorporated into the SSM library. Additionally, we observed 100% coverage of single base mutations and coverage of two and three base substitutions higher than previously reported (Table 1). The number of transformed colonies in the Pfunkel procedure did not impose a bottleneck on library complexity since the number of transformed colonies exceeded the library size by seven-fold, corresponding to a theoretical 99.9% library coverage [34]. Based on this analysis, we independently conclude that Pfunkel can produce comprehensive SSM libraries. The single-pot reaction can produce high-coverage SSM libraries in two days with minimal hands-on time.

**Table 1:** Mutagenesis statistics for different experimental conditions. Comparisons to theoretical predictions and previous literature data are shown as reference [24].

	*Theoretical*	*Firnberg et al*. *results* [24]	QuikChange Primers		Scripted Pfunkel Primers	
		Plated	Plated	Culture	Plated	Culture
Sequences (reads)		*787*,*488*	414,410	319,179	510,126	436,135
**NNN primer base composition**						
T	*25.0%*	*17.1%*	15.2%	16.7%	22.8%	19.2%
A	*25.0%*	*18.3%*	14.0%	15.7%	20.2%	19.7%
C	*25.0%*	*18.3%*	17.5%	18.5%	16.8%	19.8%
G	*25.0%*	*46.3%*	53.3%	49.0%	40.2%	41.4%
**Percent of possible codon substitiutions observed**						
1-base substitution		*99.6%*	100.0%	100.0%	100.0%	100.0%
2-base substitutions		*97.7%*	99.6%	96.4%	98.6%	83.7%
3-base substitutions		*95.3%*	98.7%	88.3%	97.3%	54.4%
All substitutions		*97.0%*	99.3%	93.5%	98.3%	73.5%
**Percent of reads with**						
No nonsynonymous mutations	*1.6%*	*26.2%*	22.4%	55.0%	62.6%	87.0%
One nonsynonymous mutation	*98.4%*	*56.7%*	70.6%	40.0%	34.1%	12.0%
Multiple nonsynonymous mutations	*0.0%*	*17.1%*	7.0%	5.0%	3.3%	1.0%
**Coverage of possible single nonsynonymous mutations**			99.9%	98.0%	99.5%	89.1%

While Pfunkel is a simple and reliable method to create high-coverage SSM libraries, the costs associated with primer synthesis are not trivial. For a protein of length *L*, the cost of the primer set is $3.90*L ($0.10 per base and 39 bases per primer) (2014, Integrated DNA Technologies, Corralville, IA). Accordingly, we looked for ways to improve the Pfunkel method by reducing method cost. **1**. Shorter primer lengths would decrease cost. Our initial primer set was designed using a QuikChange calculator that suggested longer primer lengths than the custom primer design script provided by in the Pfunkel paper. **2**. Recovering plasmid DNA in liquid culture would reduce both cost and time. In the current procedure following transformation, cells are plated on expensive BioAssay plates.

We hypothesized that shorter primers would produce SSM libraries with equally high coverage but a decreased percentage of reads containing exactly one mutation. To test this we produced a second primer set using the custom primer design script from Firnberg et al. (referred to as scripted Pfunkel primers) [24]. This primer set averaged 27 bp in length while the QuikChange primers were, on average, 39 bp. We evaluated the two primer sets using three variables that contribute to inefficient sequencing: 1) percentage of wild-type reads; 2) fractional library coverage; and 3) the number of double mutants. In comparison to the QuikChange primer set, libraries prepared with the scripted Pfunkel primers had a much higher rate of wild-type sequences (62.6%), lower library coverage (99.5%), but a lower rate of double mutants (3.3%) (Table 1). Although for the QuikChange primer set there is a higher rate of double mutants, all are accounted for in the sequencing and so do not influence later data analysis. Thus, the cost of synthesizing longer QuikChange primers is more than balanced by the benefit of a high-quality SSM library, which requires fewer DNA sequencing reads for full library coverage.

In the original Pfunkel method, following transformation cells were plated on large BioAssay plates and grown at 37°C overnight. Recovering the library in solution without plating could save cost and time. To determine whether library quality suffers without plating, two parallel Pfunkel reactions were performed with the QuikChange or Pfunkel Scripted primer sets. Following transformation, half of the cells were plated on a selective plate while the other half was grown to an OD_600_ of 0.1 in a liquid culture. Cells were then harvested, and plasmid was recovered and sequenced. Cells recovered in liquid culture showed 73–93% coverage of all possible codon substitutions, much lower than the 98.3–99.3% observed for the libraries that were plated. The liquid culture data also showed a bias against NS mutations. For example, using the QuikChange primer set and plating the cells resulted in 70.6% of the reads containing exactly one NS mutation, whereas growing cells in culture resulted in only 40.0% of reads containing one NS mutation (Table 1). Based on these experiments, we conclude that plating cells following transformation is necessary to produce high-quality SSM libraries.

Theoretically, mutational frequencies caused by saturation mutagenesis with NNN codons should be equal across bases. However, consistent with the results presented by Firnberg *et al*., we find that guanosine (G) bases are enriched relative to theoretical predictions (Table 1) [24]. Since we see very little difference in the incorporation of single bases at the DNA level between the two sets of mutagenic primers, the artificial enrichment of G bases is likely the result of improper machine mixing of the NNN mutations in primer synthesis, as previously suggested [24]. While hand mixing of the nucleotides during primer synthesis may reduce the bias, it would substantially increase primer cost. Alternatively, the enrichment of G bases could be introduced by a bias in primer annealing as suggested by Jain and Varadarajan [35]. Neverless, since in our protocols the average library member is counted at least 100 times, the observed level of bias is tolerable.

The plasmid DNA encoding the SSM library must be transformed into the host organisms used for selections. If multiple plasmids are transformed into a single cell, gain-of-function variants could potentially compensate for weaker variants. To account for this, we have derived a correction factor for the measured enrichment ratio as a function of percentage of double transformants (S3 Note). For libraries with less than 10% doubly transformed cells this correction can be neglected because its absolute magnitude correction is less than 0.35 (S1 Fig.), which is comparable to the experimental error in determining enrichment ratios from sequencing data for loss-of-function variants. However, at higher percentages of doubly transformed cells this effect may be significant (S1 Fig.) and controls must be run to minimize artifacts [36].

In our typical workflow we use *E. coli* for growth-based selections and *S. cerevisiae* for yeast display of binding proteins [37]. The most common yeast display plasmid contains a CEN6/ARSH4 ori maintaining a low plasmid copy number, such that co-transformed plasmids are segregated well before FACS [38]. For many *E. coli*-based systems however, plasmids of medium to high copy numbers do not efficiently segregate and the percentage of double transformants needs to be quantified. Goldsmith et al. suggested a strategy which we follow here [36]. The starting plasmid pJK_proJK1_kanR_LGK was modified by changing the antibiotic resistance from kanamycin to ampicillin, forming the plasmid pJK_proJK1_LGK. These two plasmids were mixed at a mass ratio of 1:100, respectively, and 40 ng of this mix was transformed into 40 μL of electrocompetent Tuner cells. The reaction was plated on ampicillin, kanamycin and ampicillin/kanamycin selective plates and grown overnight. The colonies were counted and the percentage of double transformants was calculated by taking the ratio of the number of dual antibiotic resistant colonies over the number of solely kanamycin resistant colonies (S4 Table). Under these conditions, the rate of double transformants is on the order of 2%, well below the 10% threshold. Additionally, in this specific case, we found that the number of solely ampicillin transformants is more than sufficient to support the degeneracy of a library size of 2,560. We recommend re-running this experiment for every library, as transformation conditions often vary.

### Selections

Methods for selection are chosen based on protein function. Selections should be designed such that the widest range of activity levels can be resolved. Protein binding activity is usually screened/sorted by phage, bacterial, or yeast display platforms [39–42]. The latter two methods resolve the population by FACS; in this section we derive equations governing the grouping of different variants by FACS and suggest optimal experimental parameters. We also show equations that govern the appropriate choice of experimental parameters for growth-based selections.

**Fig 3:**
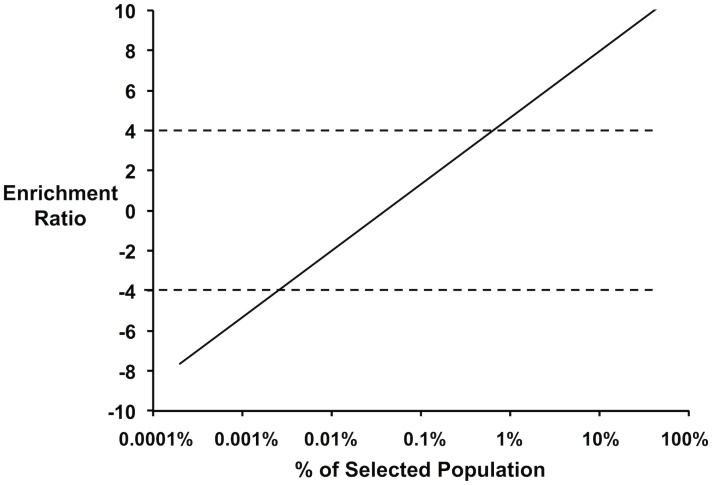
Enrichment ratio of a clone as a function of its abundance in the selected population. The dynamic range of the method lies between enrichment ratios of-4 to 4 (indicated by horizontal dashed lines) such that (i.) single clones do not dominate the selected population; and (ii.) loss-of-function clones are not completely removed from the population.

To ensure proper coverage, selections are designed such that on average there is 200–500 fold coverage of each variant in the unselected population. Sequencing to this depth requires on the order of 500,000 quality 150-bp reads. The enrichment of an individual variant is described as the log_2_ ratio of its frequency in the selected population to the unselected population. For a library containing 2,500 members and sampled at 200-fold coverage, there is a lower enrichment limit of-7.5 for mutants counted once after selection and an upper bound of 11.3 for a variant that completely overtakes the population. Because intrinsic error (Poisson noise) is lowered when the counting threshold is set much higher than 1, and because allowing a single variant to overtake the population provides no data about the remaining positions, the practical dynamic range for the selection range spans enrichment values of-4 to 4. Selections should be designed to best span this range of enrichment values. The dynamic range will vary minimally with increasing library coverage: every 2-fold increase in coverage results in decreasing the lower bound of the enrichment ratio by 1 unit. Fig. 3 shows a mutant with an enrichment ratio of-4 and makes up 0.0024% of the selected population, while a variant with an enrichment ratio of 4 and makes up 0.8% of the selected population. Among 500,000 sequence reads from the selected library, the former variant is observed 12 times and the latter 3,400 times.

**Enzymes: Growth selections**. The enrichment value (*ε*_*i*_) of an individual variant *i* depends on the average growth rate of the library population (${\overset{-}{\mu }}_{p}$), the number of doubling times the culture is allowed to grow (*g*_*p*_), and the growth rate of the individual variant (*μ*_*i*_):

$$\varepsilon_{i}=g_{p}\left(\frac{\mu_{i}}{{\bar{\mu }}_{p}}-1\right)$$ (12)

**Fig 4:**
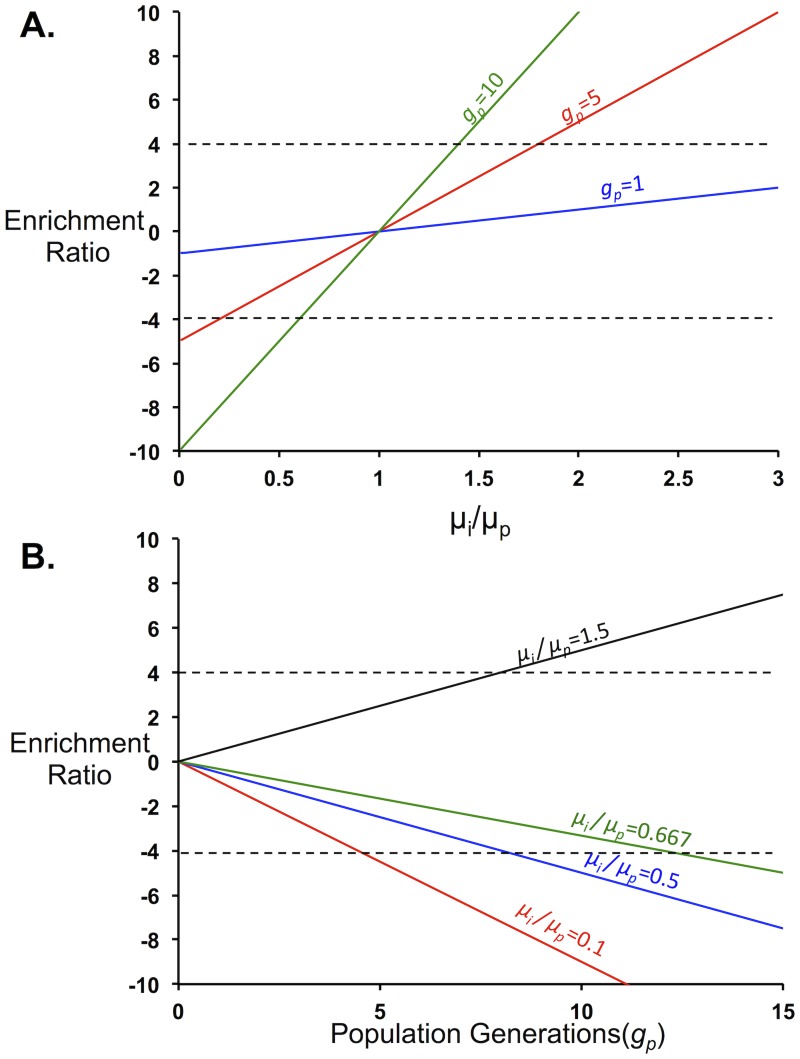
Growth Selection Parameters. The parameters of growth-based selections should be chosen such that the range of enrichment ratios for the population lies between-4 and 4. *A*. Enrichment ratios as a function of the individual growth rate compared to the population growth rate. Following one generation of population growth (blue) the enrichment ratios remain around zero. Increasing the number of population generations the experiment is allowed to grow (1 generation, blue, 5 generations, red and 10 generations, green) increases the experimental resolution in discriminating mutant growth phenotypes. *B*. Enrichment ratios as a function of average population generation (g_p_) for various $\frac{\mu_{i}}{\mu_{p}}$. For $\frac{\mu_{i}}{\mu_{p}}$ values less than one (0.1, red; 0.5, blue; and 0.667, green) the enrichment ratios decrease with increasing population generations. Variants with values $\frac{\mu_{i}}{\mu_{p}}$ values above one (1.5, black) show enhanced enrichment with increasing population generations.

Growth selections should be designed such that the number of generations the culture is allowed to grow fits a reasonable time frame (under 2 days) and there is high resolution of fitness for the entire library. Fig. 4 shows the enrichment ratios for a range of specific growth rates relative to the population-averaged growth rate for different numbers of doubling times. According to these results, the dynamic range of protein activities is maximized between five and ten doubling times. This range allows resolution of all variants with growth rates above 0.2 of the population-averaged growth rate. Furthermore, limiting the number of doublings minimizes the effect of spontaneous mutations in the background strain [43].

**Protein Binders/Transcriptional Regulators/Membrane Proteins: FACS Screens**. FACS is used in many different screening scenarios including protein binding, transcriptional activation, gene silencing, and localization studies [9,10,44–46]. In each of these screens the presence of cellular fluorescence corresponds to some underlying protein activity. In yeast display, the binding affinity of a given protein-protein or protein-small molecule interaction is assessed by binding of a biotinylated protein or small molecule (present at a concentration near the dissociation constant for the interaction) to a surface-displayed protein, followed by labeling with fluorescently-conjugated streptavidin [37]. In this case, higher fluorescence indicates increased binding affinity for the biotinylated protein.

**Fig 5:**
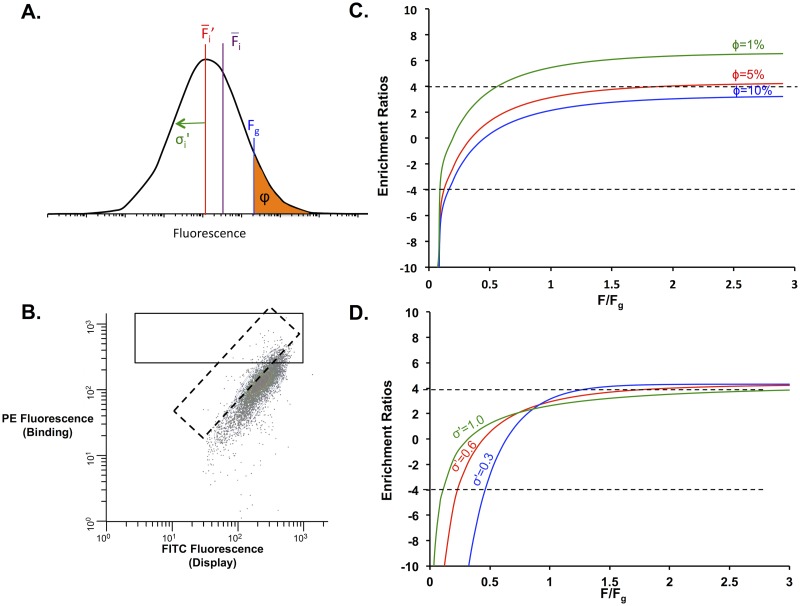
FACS Selection Parameters. *A*. Individual fluorescence from a clonal population of cells is log-normally distributed with a log-transformed standard deviation σ´, log-transformed mean fluorescence ${\overset{-}{F}}_{i}{\;}^{\prime }$ and mean fluorescence${\overset{-}{F}}_{i}$. Cells are collected based on the gating fluorescence, *F*_*g*_, which controls the fraction of cells collected, φ. *B*. Sample FACS readout for yeast-surface display. The x-axis represents the fluorescence of the displayed population, whereas the y-axis represents the fluorescence of the binding activity of interest. Both square (solid line) and diagonal (dashed line) gates can be drawn around the population to be sorted. Diagonal gates will decrease the standard deviation of the transformed fluorescence distribution, narrowing the range of protein activities that can be resolved. *C*. The enrichment values as a function of the ratio of the individual fluorescence to the gating fluorescence for different gating percentages (1%, green; 5%, red; 10%, blue) for σ´ = 0.6. In more stringent sorts, resolution is lost in the enrichment ratios for poor binders. The dynamic range for the fraction of cells collected is between 5 and 10%. *D*. The enrichment values as a function of the ratio of the individual fluorescence to the gating fluorescence for different standard deviations (1.0, green; 0.6, red; 0.3, blue) for a gating percentage of 5%. Setting smaller standard deviations by two-color sorting using diagonal gates narrows the dynamic range of enrichment values for the sorted populations.

The distribution of fluorescent intensity for individual cells is log normally distributed (Fig. 5A) with a mean fluorescence ${\overset{-}{F}}_{i}{\;}^{\prime }$and a clone-independent standard deviation *σ´*. To sort populations, square (normal to one axis) or diagonal gates (normalizing for surface expression) are usually drawn (Fig. 5B); these gates sort a specific fraction of the population, *ϕ*, that exceeds a gating fluorescence, *F*_*g*_. Sorting cells using one-color (square gate) is most common. However, two-color sorting (diagonal gating) is often used to correct for intrinsic noise caused by distributions in cell size, among other factors [47]. Similarly, two-color sorting can be used in protein display techniques to normalize for cell-to-cell variation in surface expression [48]. Two-color sorting results in a log normal distribution for the transformed fluorescence but with a significantly reduced standard deviation. As such, these sorts can be described by the FACS equations derived in the Theory section. Gating the top fraction of the fluorescent distribution enriches the sorted population in variants with enhanced activity. The enrichment ratio of a single clone can be described by rearranging Equation (18):

$$\varepsilon_{i}=\log_{2}\left[\frac{\left(1-erf\left(\frac{\frac{\sigma^{'}}{2}+\ln\frac{F_{g}}{F_{i}}}{\sigma^{'}\sqrt{2}}\right)\right)}{\phi }\right]-1$$ (21)

where *ε*_*i*_ is the enrichment ratio of an individual clone, σ*´* is the standard deviation of the single-clone log-normal fluorescence distribution, ${\overset{-}{F}}_{i}$is the mean fluorescence of an individual mutant, *F*_*g*_ is the gating fluorescence and *ϕ* is the gating percentage (Fig. 5A). *σ´* can be calculated independently for a clonal population of the starting variant using the fluorescence distribution.

To determine optimal sorting parameters, we have plotted enrichment ratios as a function of the ratio of individual fluorescence to the gating threshold for fluorescence $\left(\frac{{\bar{F}}_{i}}{{\bar{F}}_{wt}}\right)$ at different gating percentages (Fig. 5C). It should be noted that the gating threshold is dependent on the fraction of cells that will be collected. A less-stringent gate, with *ϕ* equal to 10%, provides a wide distribution of enrichment values for many of the clonal populations but will not resolve differences in binding above $\left(\frac{{\bar{F}}_{i}}{F_{g}}\right)$>1.3. A stringent gate at *ϕ =* 1% enriches strong binders to a ratio of about 6, providing little information about poor binders. We find the optimal *ϕ* to be around 5%, where the enrichment ratios of both poor binders and strong binders (relative to the original binding interaction) fall within the dynamic range of 4 to -4 (Fig. 5C). FACS selections should be designed such that the ratio of the fluorescence for the starting construct relative to the anticipated gating threshold is less than 0.5. The actual gating threshold *F*_*g*_, however, is governed by *ϕ*, the percentage of the cells that will be collected. Another parameter that can be modified is the log-transformed standard deviation of the fluorescent distribution. For example, this standard deviation can be decreased by drawing a diagonal gate so that the populations are sorted by two fluorescent parameters, which compensates for certain sources of noise. Fig. 5D shows the enrichment ratio of clones as a function of the ratio of individual fluorescence to the gating fluorescence at a single gating fluorescence for different standard deviations. Populations with a smaller standard deviation show a smaller range for collection than those with a larger standard deviation. It is recommended that for applications where elucidation of gain-of-function and loss-of-function variants is desired, a square gate should be used. However, a diagonal gate should be used for enriching the population to uncover mostly improved variants.

Finally, in the specific case of yeast surface display of protein binders we label the displayed proteins at levels approximately half of the dissociation constant for the starting protein-ligand interaction. Optimal labeling concentrations can be calculated using parameters set by Boder and Wittrup [49]. Higher activity variants are often isolated using multiple sorts from yeast display or other display-based methods. However, our normalization equations only allow quantitative comparisons between populations occurring during a single sort. Theoretically using one sort is sufficient to resolve most of the population while minimizing time and down stream processing for FACS. As necessary, further sorting can be done to finely discriminate among the enhanced binding variants (Fig. 4C). In the specific case of yeast surface display, the labeling concentration for the second sort can be set at a much lower level than the first sort. Analysis of the population frequencies after the second sort compared to the first sort can be done using the same normalization equations as above.

### Deep Sequencing Library Preparation

**Fig 6:**
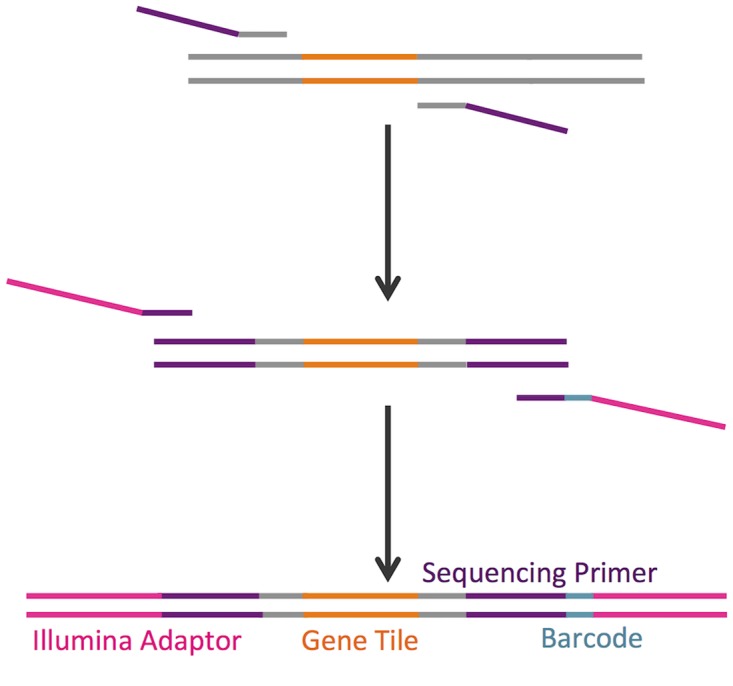
2-step PCR method for deep sequencing preparation of libraries. PCR reactions are shown for two separate gene tiles containing single mutations (orange and green). Primers are designed to be complementary to flanking regions (grey) of each tile, with encoded single mutations. The first set of primers includes the flanking regions and Illumina sequencing primers (purple). In the next step, outer primers add the Illumina adaptor (pink) and multiplexing index (teal) sequences to the gene. The PCR reaction is performed in a single tube using a 1:2 molar ratio of inner to outer primers and bead purified to remove primer dimer products. The purified library is ready for sequencing without further modifications. While the first set of primers is specific to a single gene tile, the outer primer set is universal.

Deep sequencing was used to obtain count data of each variant in the population using an Illumina MiSeq in 150-bp paired end mode. Plasmid DNA was extracted using a Qiagen miniprep kit (for *E. coli*) or a modified smash and grab protocol (for *S. cerevisiae*; see S4 Note). Following plasmid extraction, a modular two-step PCR method was used to amplify the gene tile and to add the Illumina sequencing, adaptor, and barcode sequences (Fig. 6). The two-step PCR procedure involves two sets of primers. The first, inner, set amplifies out the gene tile using the gene sequence up- and downstream of the tile and attaches a segment of the sequencing primer. Inner primers are specific to each tile and can be designed using a custom script (S2 Script). The outer primers attach the Illumina adaptors and a barcode on the 3’ end of the gene. These primers are a universal set and can be used across different experiments (full sequences listed in S2 Table).

**Fig 7:**
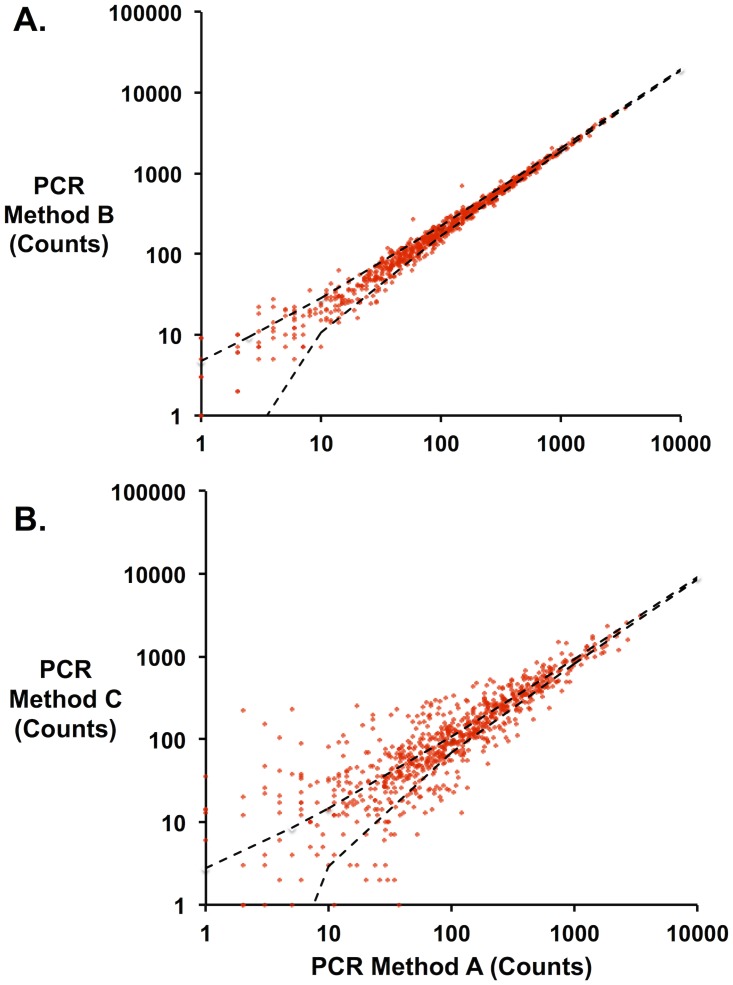
Errors introduced by different PCR methods. Identical mutant libraries were prepared for sequencing using three different PCR methods. The number of counts for each library member was compared across the different methods. Each point represents a specific mutant sequence. For each panel, dashed black lines represent the 95% confidence interval for the Poisson noise between different methods. *A*. Method B v. Method A. Above 10 counts, the data fall almost completely within minimal error predicted by Poisson noise. *B*. By contrast, Method C shows significant variance in counts relative to Method A.

Three different PCR methods (Methods A, B, and C, S3 Table) were used to attach both sets of primers to an unselected library of LGK variants, and frequencies of each variant were quantified by deep sequencing. If there were no differences in PCR bias among methods, the error in calculating the normalized amount (frequency) of each variant in the population would approach the Poisson limit. Comparisons of variant frequency between Method A and Method B show error between methods approaching this theoretical minimum (Fig. 7A). By contrast, Method C shows much larger differences with respect to Method A (Fig. 7B) indicating a bias in the PCR method. S2 Fig. shows the protein mutation distribution compared to the theoretical coverage for this unselected library following preparation for sequencing by each of the different methods. Amino acid mutations are enriched in proline (CCN), alanine (GCN), histidine (CAT, CAC) and arginine (CGN) most likely because of the overrepresentation of G bases in the NNN codons in the primer set as discussed above. While proline is grossly overrepresented in the library as a consequence (20% reads vs. 5% from theoretical expectation), this bias is tolerable because of oversampling of population members in sequencing. From these results we recommend Method A as it requires the least amount of hands-on and setup time.

### Normalization and Data Analysis

The frequency of individual variants in selected and unselected populations is extracted from raw sequencing files using the Enrich software suite [26]. Briefly, the forward and reverse reads are aligned, errors between reads are resolved, and the combined sequence is aligned to the starting DNA sequence. Each mutation is recorded and counted, these counts are normalized to frequencies, and the enrichment ratios are found by comparison of the frequency of a given mutant in the selected to the unselected population. To facilitate comparisons of variants across different selection conditions, we have derived normalization equations that transform these enrichment ratios (*ε*_*i*_) to an objective fitness metric. If a variant is not present in the unselected library then we are unable to determine the fitness metric for that variant.

For growth-based selections, this fitness metric is defined as:

$$\zeta_{i}=\log_{2}\left(\frac{\frac{\varepsilon_{i}}{g_{p}}+1}{\frac{\varepsilon_{wt}}{g_{}}+1}\right)$$ (11)

This metric requires two additional pieces of information. First, the enrichment ratio of the starting or reference sequence must be known (*ε*_*wt*_). Fortunately, this reference variant is generally present in the library, regenerated at each position by the appropriate NNN primer. Second, the number of doubling periods (*g_p_*) for the culture must be calculated from the initial and final optical cell density.

**Fig 8:**
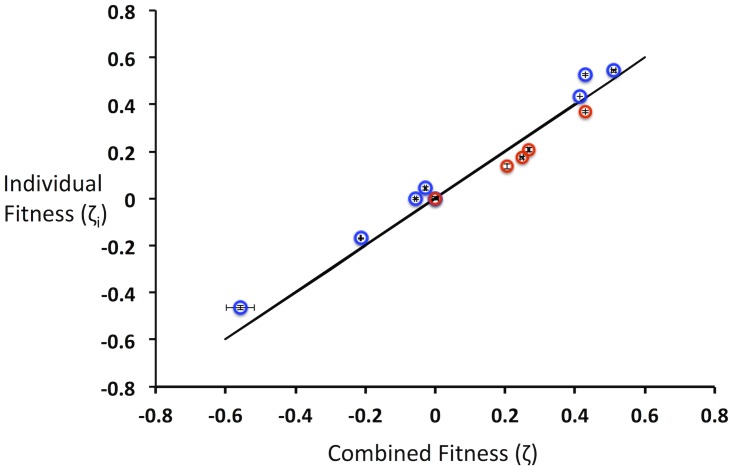
Experimental validation of growth rate normalization relation. *E. coli* harboring 11 different plasmids driving differential eGFP levels were grown in a single population or in two separate populations. The fitness from the separate populations (represented by blue and red open circles) and combined populations were evaluated and compared. Error bars represent one standard deviation from two independent experiments. The solid black line represents the theoretically ideal relationship between individual and combined fitness.

To determine whether this relation could reproduce the fitness of individual variants in different populations, we grew populations of *E. coli* harboring plasmids expressing different levels of eGFP expression [22]. Differential expression results in growth differences among individual strains of nearly 2-fold (n = 11; range 0.46 ≤ *μ*_*i*_ ≥ 0.76 h^-1^). Initially, we mixed these variants into a single population and determined individual fitness values after 8.6 average population doublings. Then, we mixed subsets of these variants into two different populations and again determined individual fitness values. Ideally, these fitness values would be exactly the same across the different populations. A best-fit regression line of the fitness values for individuals compared across the different populations gives a slope of 1.04 (R^2^ = 0.96), very close to the ideal case of 1 (Fig. 8). Based on these results, we conclude that the derived relation is an effective way to normalize the fitness of individual mutants across different populations, thus allowing quantitative comparisons across different selected populations.

In the case of FACS, fitness can be measured across populations according to the following relation:

$$\zeta_{i}={log}_{2}[e\sqrt{2}\;\sigma^{\prime }(erf^{-1}(1-\phi 2^{\varepsilon_{wt}+1})-erf^{-1}(1-\phi 2^{\varepsilon_{i}+1}))]$$ (20)

Thus, the fitness of an individual variant *ζ*_*i*_ can be derived from its enrichment ratio (*ε*_*i*_) given the enrichment ratio of the wild type (*ε*_*wt*_), the percentage of the entire population collected under the sorting gates (*ϕ*), and a log_2-_transformed standard deviation of fluorescence for a variant (*σ′*). Since this standard deviation is a scalar in the function, rank ordering of fitness across different populations can be done without directly measuring this quantity. By contrast, the gating percentage of the library is easily measured. Thus, experimentally measured parameters, combined with this relation, allow unambiguous comparisons of variants across different populations.

## Conclusions

In this paper we have presented a standardized method for producing the sequence function determinants for entire protein sequences. Furthermore, we have derived equations that allow users to identify optimal selection conditions for their target of interest and to directly compare variants across different populations. Using this method, users can create functional landscapes for full-length genes quickly and efficiently. These landscapes can be applied to protein engineering, for antibody-epitope mapping, and for many different end uses. Additionally, these landscapes can be integrated with computational design methods, either by highlighting existing shortcomings of computational prediction software or as experimental data to guide computational trajectories in search algorithms [50].

The best practices and a step-by-step protocol governing each step in the process are listed in S2 Note. These guidelines add to the body of literature for recent sequence-function mapping protocols [13–15,51,52]. Notably, many of the individual steps presented here are fully compatible with, and can enhance, these other published protocols. For example, the general fitness equations derived for growth-based selections can be used to optimize experimental set-up for the EMPIRIC approach [15]. Additionally, the general gene tiling and primer design strategy can be applied for assessing full-length sequences with EMPIRIC.

Because of the gene tiling approach, there is no practical upper limit on a gene sequence to be tested. In principle, this approach can be applied to targets much larger than single gene products like complete metabolic pathways. One downside of current approaches is that short read lengths inherent in existing sequencing platforms limit libraries to single mutants or coupled mutants that are proximal in a contiguous stretch of the gene. Resolving this limitation requires new sequencing methods able to resolve long reads with very low error rates. In the near future, perhaps sequencing-function mapping of multiple simultaneous mutations can be used as a way to fine tune cooperation effects between different beneficial mutations or neutral mutations identified from a single-site saturation mutagenesis library.

## Supporting Information

S1 Fig Enrichment correction factor from double transformation artifacts. The enrichment correction factor is characterized by the true enrichment ratio (ε_t_) minus the measured enrichment ratio (ε_m_) for different, variant growth rates relative to the population growth rate. *A*. The distribution of the individual growth rates in the population was assumed to be a bimodal-guassian with means of μ_i_/μ_p_ = 0.2 and 0.9 and a standard deviation of 0.06, broadly consistent with individual variant growth rates observed for a library. *B*. Here the assumed double transformation rate is assumed to be 10% and the correction factor is plotted for different numbers of population doubling periods. (2 red, 5 blue, 8 black,10 green). *C*. Here the assumed population doubling periods is 8 and the correction factor is plotted for different double transformation rates. If the double transformation rate is less than 10% and the population doubling periods is about 8 the correction factor is negligible and does not need to be considered.
(TIFF)

S2 Fig Library Amino Acid Composition and PCR Bias Determination. The distribution of incorporated amino acids was used to determine any bias introduced by PCR methods to prepare the library for deep sequencing. The frequency of each mutation overall in the 40 residue region was compared to the theoretical frequency (orange) for each residue type. Method A (red) and Method B (blue) show little difference between the distribution of amino acid substitutions, while Method C (green) shows slight differences at some residue types. The artificial enrichment in proline, alanine and histidine occurs because degenerate primers used for mutagenesis contained an overabundance of guanine bases. The different PCR methods do not show a specific bias toward any single residue.
(TIFF)

S1 Table pJK_eGFP Series Plasmids. pJK-series plasmids driving eGFP expression. Each plasmid differs only in the strength of the promoter sequence. Specific base-pair differences at the-35 hexamer compared to the base promoter proB are shown in red. Enrichment ratios (ε) for different growth experiments are recorded. Recorded errors are listed as one standard deviation from two independent experiments.
(DOCX)

S2 Table Primer Sequences. Primer sequences for deep sequencing preparation.-LGK and GFP-named sequences are the inner primers (gene-specific primers) consisting of gene overlap (black) and Illumina sequencing primer (green). RPI-series primers are derived partially from the Illumina RNA TruSeq preparation kit. These primer sequences contain an Illumina sequencing primer (green), a barcode (blue) in the reverse direction, and an Illumina adaptor sequence (red). Barcoded sequences are used to multiplex the sequencing reactions.
(DOCX)

S3 Table PCR Reaction Conditions. Conditions tested in the library DNA preparation for Illumina sequencing.
(DOCX)

S4 Table Determination of double transformation percentage. Results from double transformation experiments.
(DOCX)

S1 Note Plasmid Maps for pJK-series plasmids. (DOCX)

S2 Note Practical Considerations for High-Resolution Sequence-Function Mapping. (DOCX)

S3 Note Theoretical effects of double transformation on enrichment ratios for growth-based selections. (DOCX)

S4 Note Protocol for Plasmid Extraction and Deep Sequencing Preparation of Yeast-based plasmids. (DOCX)

S1 Script EnrichPatch.py. (PY)

S2 Script GeneTile.m. (M)

S1 File S2 Script Sample File. (TXT)
