# Supplementary figures and images for: High-Resolution Sequence-Function Mapping of Full-Length Proteins
Source: PLoS One. 2015 Mar 19;10(3):e0118193. doi: 10.1371/journal.pone.0118193 (PMC4366243; doi:10.1371/journal.pone.0118193)

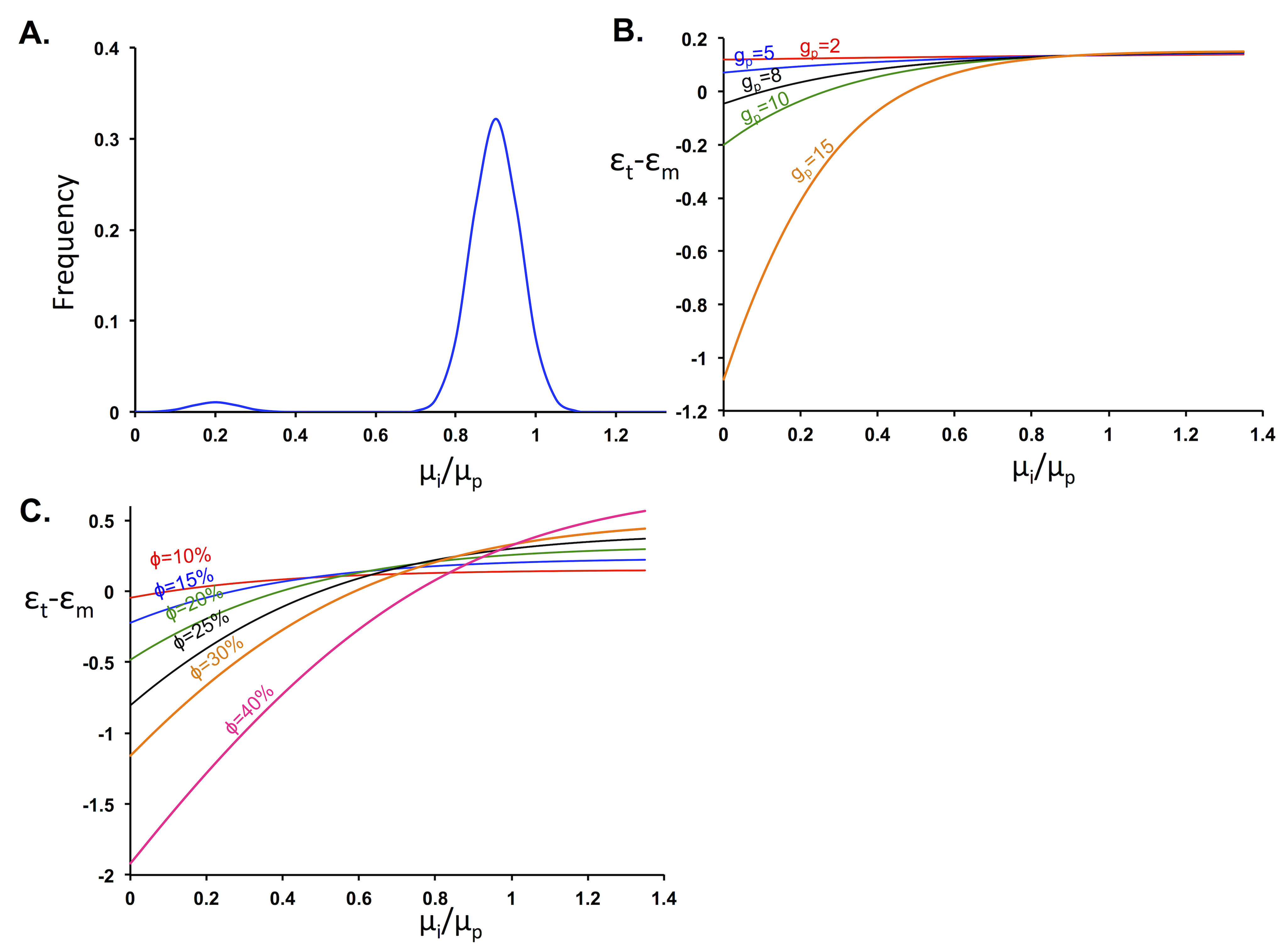

Supplement: S1 Fig — The enrichment correction factor is characterized by the true enrichment ratio (εt) minus the measured enrichment ratio (εm) for different, variant growth rates relative to the population growth rate. A. The distribution of the individual growth rates in the population was assumed to be a bimodal-guassian with means of μi/μp = 0.2 and 0.9 and a standard deviation of 0.06, broadly consistent with individual variant growth rates observed for a library. B. Here the assumed double transformation rate is assumed to be 10% and the correction factor is plotted for different numbers of population doubling periods. (2 red, 5 blue, 8 black,10 green). C. Here the assumed population doubling periods is 8 and the correction factor is plotted for different double transformation rates. If the double transformation rate is less than 10% and the population doubling periods is about 8 the correction factor is negligible and does not need to be considered. (TIFF) [file pone.0118193.s001.tiff]

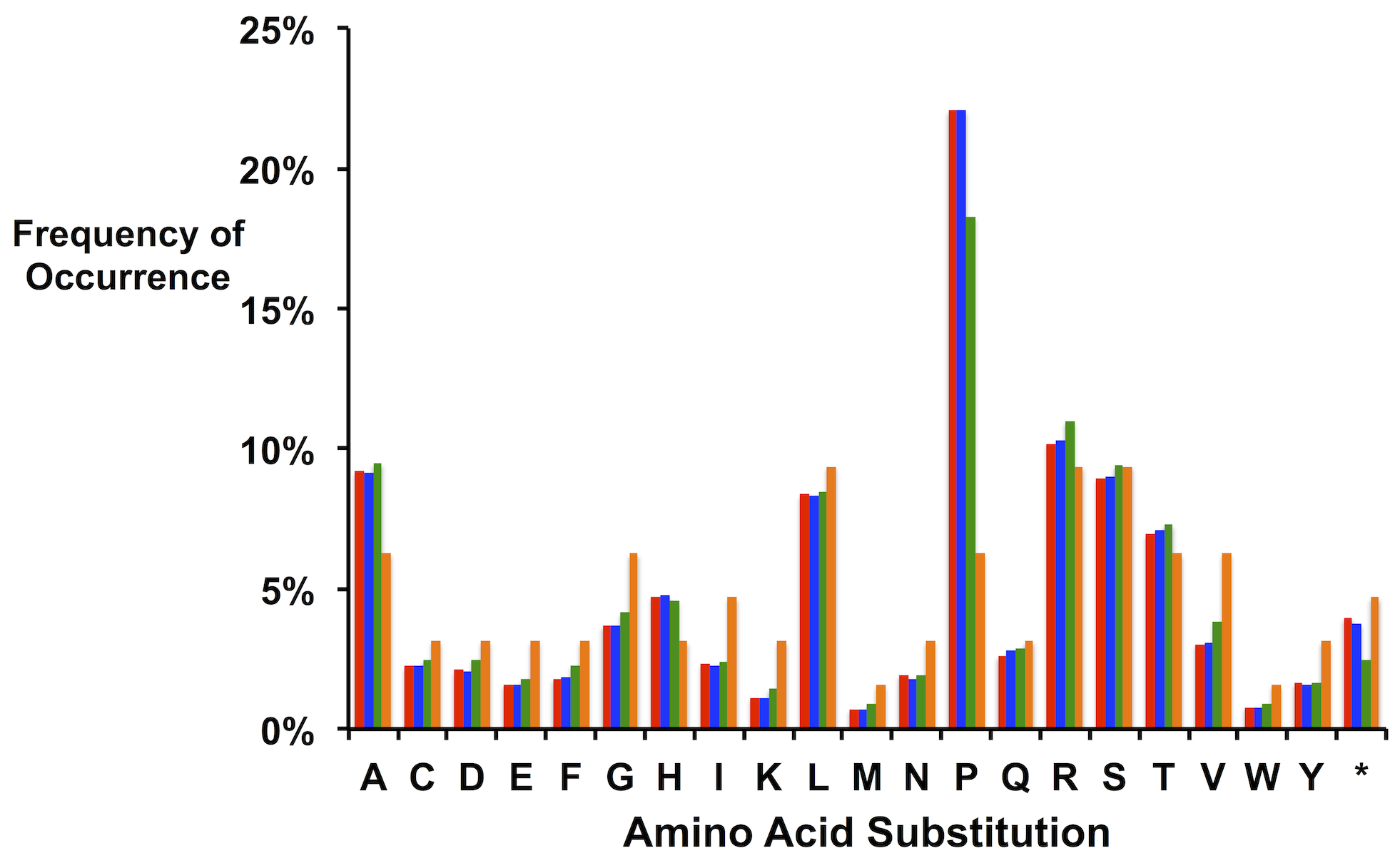

Supplement: S2 Fig — The distribution of incorporated amino acids was used to determine any bias introduced by PCR methods to prepare the library for deep sequencing. The frequency of each mutation overall in the 40 residue region was compared to the theoretical frequency (orange) for each residue type. Method A (red) and Method B (blue) show little difference between the distribution of amino acid substitutions, while Method C (green) shows slight differences at some residue types. The artificial enrichment in proline, alanine and histidine occurs because degenerate primers used for mutagenesis contained an overabundance of guanine bases. The different PCR methods do not show a specific bias toward any single residue. (TIFF) [file pone.0118193.s002.tiff]
