# Supplementary material for: High-Resolution Sequence-Function Mapping of Full-Length Proteins
Source: PLoS One. 2015 Mar 19;10(3):e0118193. doi: 10.1371/journal.pone.0118193 (PMC4366243; doi:10.1371/journal.pone.0118193)
Supplement: S1 Table — pJK-series plasmids driving eGFP expression. Each plasmid differs only in the strength of the promoter sequence. Specific base-pair differences at the-35 hexamer compared to the base promoter proB are shown in red. Enrichment ratios (ε) for different growth experiments are recorded. Recorded errors are listed as one standard deviation from two independent experiments. (DOCX) [file pone.0118193.s003.docx]

**Table S1: pJK_eGFP Series Plasmids.** pJK-series plasmids driving eGFP expression. Each plasmid is differentdiffers only in the strength of the promoter sequence. Specific base-pair differences at the -35 hexamer compared to the base promoter proB are shown in red. Enrichment ratios (ε) for different growth experiments are recorded. Recorded errors are listed as one standard deviation from two independent experiments.

| Promoter | -35 hexamer | -10 hexamer | ε - combined | ε - population #1 | ε - population# 2 |
| --- | --- | --- | --- | --- | --- |
| **proB** | **TTTACG** | **TAATAT** | -1.18 ± 0.00 | -0.93 ± 0.04 | -1.31 ± 0.04 |
| proK21 | TTTACA | TAATAT | 1.40 ± 0.01 | 2.44 ± 0.03 | 0.80 ± 0.03 |
| proNR2 | CTTACG | TAATAT | 0.21 ± 0.03 | N/A | -0.37 ± 0.04 |
| proK16 | TTTTCG | TAATAT | 1.98 ± 0.05 | 2.57 ± 0.05 | N/A |
| proK14 | TGTACG | TAATAT | 0.34 ± 0.02 | N/A | -0.18 ± 0.06 |
| proK11 | TTTAGT | TAATAT | -0.04 ± 0.00 | N/A | -0.59 ± 0.06 |
| proK9 | TTTACC | TAATAT | -1.32 ± 0.02 | -0.70 ± 0.04 | N/A |
| proK6 | TTGTCG | TAATAT | -1.46 ± 0.02 | -0.94 ± 0.02 | N/A |
| proK2 | TTGCCG | TAATAT | -2.20 ± 0.01 | -1.78 ± 0.01 | N/A |
| proNR1 | TTTAAC | TAATAT | 1.29 ± 0.00 | 1.76 ± 0.01 | N/A |
| proK1 | TTTACT | TAATAT | -3.56 ± 0.14 | -3.04 ± 0.03 | N/A |
