# Supplementary material for: High-Resolution Sequence-Function Mapping of Full-Length Proteins
Source: PLoS One. 2015 Mar 19;10(3):e0118193. doi: 10.1371/journal.pone.0118193 (PMC4366243; doi:10.1371/journal.pone.0118193)
Supplement: S2 Table — Primer sequences for deep sequencing preparation.-LGK and GFP-named sequences are the inner primers (gene-specific primers) consisting of gene overlap (black) and Illumina sequencing primer (green). RPI-series primers are derived partially from the Illumina RNA TruSeq preparation kit. These primer sequences contain an Illumina sequencing primer (green), a barcode (blue) in the reverse direction, and an Illumina adaptor sequence (red). Barcoded sequences are used to multiplex the sequencing reactions. (DOCX) [file pone.0118193.s004.docx]

## Table S2. Primer Sequences

Primer sequences for deep sequencing preparation. -LGK and GFP-named sequences are the inner primers (gene-specific primers) consisting of gene overlap (black) and Illumina sequencing primer (green). RPI-series primers are derived partially from the Illumina RNA TruSeq preparation kit. These primer sequences contain an Illumina sequencing primer (green), a barcode (blue) in the reverse direction, and an Illumina adaptor sequence (red). Barcoded sequences are used to multiplex the sequencing reactions.

| **Name** | **Sequence** |
| --- | --- |
| LGK1_F | GTTCAGAGTTCTACAGTCCGACGATCTTAACTTTAAGAAGGAGATATACAT |
| LGK1_R | CCTTGGCACCCGAGAATTCCATCCATCGGAGCATC |
| GFP_F | GTTCAGAGTTCTACAGTCCGACGATCAGCTAACACCACGTCG |
| GFP_R | CCTTGGCACCCGAGAATTCCATATTTCTAGAAAGTTAAACAAAATT |
| RPI_F | AATGATACGGCGACCACCGAGATCTACACGTTCAGAGTTCTACAGTCCGA |
| RPI1 | CAAGCAGAAGACGGCATACGAGATCGTGATGTGACTGGAGTTCCTTGGCACCCGAGAATTCCA |
| RPI2 | CAAGCAGAAGACGGCATACGAGATACATCGGTGACTGGAGTTCCTTGGCACCCGAGAATTCCA |
| RPI3 | CAAGCAGAAGACGGCATACGAGATGCCTAAGTGACTGGAGTTCCTTGGCACCCGAGAATTCCA |
| RPI4 | CAAGCAGAAGACGGCATACGAGATTGGTCAGTGACTGGAGTTCCTTGGCACCCGAGAATTCCA |
| RPI5 | CAAGCAGAAGACGGCATACGAGATCACTGTGTGACTGGAGTTCCTTGGCACCCGAGAATTCCA |
| RPI6 | CAAGCAGAAGACGGCATACGAGATATTGGCGTGACTGGAGTTCCTTGGCACCCGAGAATTCCA |
| RPI7 | CAAGCAGAAGACGGCATACGAGATGATCTGGTGACTGGAGTTCCTTGGCACCCGAGAATTCCA |
| RPI8 | CAAGCAGAAGACGGCATACGAGATTCAAGTGTGACTGGAGTTCCTTGGCACCCGAGAATTCCA |
| RPI9 | CAAGCAGAAGACGGCATACGAGATCTGATCGTGACTGGAGTTCCTTGGCACCCGAGAATTCCA |
| RPI10 | CAAGCAGAAGACGGCATACGAGATAAGCTAGTGACTGGAGTTCCTTGGCACCCGAGAATTCCA |
| RPI11 | CAAGCAGAAGACGGCATACGAGATGTAGCCGTGACTGGAGTTCCTTGGCACCCGAGAATTCCA |
| RPI12 | CAAGCAGAAGACGGCATACGAGATTACAAGGTGACTGGAGTTCCTTGGCACCCGAGAATTCCA |
| RPI13 | CAAGCAGAAGACGGCATACGAGATTTGACTGTGACTGGAGTTCCTTGGCACCCGAGAATTCCA |
| RPI14 | CAAGCAGAAGACGGCATACGAGATGGAACTGTGACTGGAGTTCCTTGGCACCCGAGAATTCCA |
| RPI15 | CAAGCAGAAGACGGCATACGAGATTGACATGTGACTGGAGTTCCTTGGCACCCGAGAATTCCA |
| RPI16 | CAAGCAGAAGACGGCATACGAGATGGACGGGTGACTGGAGTTCCTTGGCACCCGAGAATTCCA |
| RPI17 | CAAGCAGAAGACGGCATACGAGATCTCTACGTGACTGGAGTTCCTTGGCACCCGAGAATTCCA |
| RPI18 | CAAGCAGAAGACGGCATACGAGATGCGGACGTGACTGGAGTTCCTTGGCACCCGAGAATTCCA |
| RPI19 | CAAGCAGAAGACGGCATACGAGATTTTCACGTGACTGGAGTTCCTTGGCACCCGAGAATTCCA |
| RPI20 | CAAGCAGAAGACGGCATACGAGATGGCCACGTGACTGGAGTTCCTTGGCACCCGAGAATTCCA |
| RPI21 | CAAGCAGAAGACGGCATACGAGATCGAAACGTGACTGGAGTTCCTTGGCACCCGAGAATTCCA |
| RPI22 | CAAGCAGAAGACGGCATACGAGATCGTACGGTGACTGGAGTTCCTTGGCACCCGAGAATTCCA |
| RPI23 | CAAGCAGAAGACGGCATACGAGATCCACTCGTGACTGGAGTTCCTTGGCACCCGAGAATTCCA |
| RPI24 | CAAGCAGAAGACGGCATACGAGATGCTACCGTGACTGGAGTTCCTTGGCACCCGAGAATTCCA |
| RPI25 | CAAGCAGAAGACGGCATACGAGATATCAGTGTGACTGGAGTTCCTTGGCACCCGAGAATTCCA |
| RPI26 | CAAGCAGAAGACGGCATACGAGATGCTCATGTGACTGGAGTTCCTTGGCACCCGAGAATTCCA |
| RPI27 | CAAGCAGAAGACGGCATACGAGATAGGAATGTGACTGGAGTTCCTTGGCACCCGAGAATTCCA |
| RPI28 | CAAGCAGAAGACGGCATACGAGATCTTTTGGTGACTGGAGTTCCTTGGCACCCGAGAATTCCA |
| RPI29 | CAAGCAGAAGACGGCATACGAGATTAGTTGGTGACTGGAGTTCCTTGGCACCCGAGAATTCCA |
| RPI30 | CAAGCAGAAGACGGCATACGAGATCCGGTGGTGACTGGAGTTCCTTGGCACCCGAGAATTCCA |
| RPI31 | CAAGCAGAAGACGGCATACGAGATATCGTGGTGACTGGAGTTCCTTGGCACCCGAGAATTCCA |
| RPI32 | CAAGCAGAAGACGGCATACGAGATTGAGTGGTGACTGGAGTTCCTTGGCACCCGAGAATTCCA |
| RPI33 | CAAGCAGAAGACGGCATACGAGATCGCCTGGTGACTGGAGTTCCTTGGCACCCGAGAATTCCA |
| RPI34 | CAAGCAGAAGACGGCATACGAGATGCCATGGTGACTGGAGTTCCTTGGCACCCGAGAATTCCA |
| RPI35 | CAAGCAGAAGACGGCATACGAGATAAAATGGTGACTGGAGTTCCTTGGCACCCGAGAATTCCA |
| RPI36 | CAAGCAGAAGACGGCATACGAGATTGTTGGGTGACTGGAGTTCCTTGGCACCCGAGAATTCCA |
| RPI37 | CAAGCAGAAGACGGCATACGAGATATTCCGGTGACTGGAGTTCCTTGGCACCCGAGAATTCCA |
| RPI38 | CAAGCAGAAGACGGCATACGAGATAGCTAGGTGACTGGAGTTCCTTGGCACCCGAGAATTCCA |
| RPI39 | CAAGCAGAAGACGGCATACGAGATGTATAGGTGACTGGAGTTCCTTGGCACCCGAGAATTCCA |
| RPI40 | CAAGCAGAAGACGGCATACGAGATTCTGAGGTGACTGGAGTTCCTTGGCACCCGAGAATTCCA |
| RPI41 | CAAGCAGAAGACGGCATACGAGATGTCGTCGTGACTGGAGTTCCTTGGCACCCGAGAATTCCA |
| RPI42 | CAAGCAGAAGACGGCATACGAGATCGATTAGTGACTGGAGTTCCTTGGCACCCGAGAATTCCA |
| RPI43 | CAAGCAGAAGACGGCATACGAGATGCTGTAGTGACTGGAGTTCCTTGGCACCCGAGAATTCCA |
| RPI44 | CAAGCAGAAGACGGCATACGAGATATTATAGTGACTGGAGTTCCTTGGCACCCGAGAATTCCA |
| RPI45 | CAAGCAGAAGACGGCATACGAGATGAATGAGTGACTGGAGTTCCTTGGCACCCGAGAATTCCA |
| RPI46 | CAAGCAGAAGACGGCATACGAGATTCGGGAGTGACTGGAGTTCCTTGGCACCCGAGAATTCCA |
| RPI47 | CAAGCAGAAGACGGCATACGAGATCTTCGAGTGACTGGAGTTCCTTGGCACCCGAGAATTCCA |
| RPI48 | CAAGCAGAAGACGGCATACGAGATTGCCGAGTGACTGGAGTTCCTTGGCACCCGAGAATTCCA |
