# Supplementary material for: High-Resolution Sequence-Function Mapping of Full-Length Proteins
Source: PLoS One. 2015 Mar 19;10(3):e0118193. doi: 10.1371/journal.pone.0118193 (PMC4366243; doi:10.1371/journal.pone.0118193)
Supplement: S3 Table — Conditions tested in the library DNA preparation for Illumina sequencing. (DOCX) [file pone.0118193.s005.docx]

**Table S3.** Conditions tested in the library DNA preparation for Illumina sequencing.

|  | Method A | Method B | Method C |
| --- | --- | --- | --- |
| Reaction Conditions  Per 50µL rxn | 10µL 5x Phusion Buffer  1µL 10mM dNTPs  2.5µL 5µM inner forward primer  2.5µL 5µM inner reverse primer  2.5µL 10µM outer forward primer  2.5µL 10µM outer reverse primer  0.5µL Phusion HF Polymerase  1ng template  Water to 50µL | 10µL 5x Phusion Buffer  1µL 10mM dNTPs  2.5µL 10µM inner forward primer  2.5µL 10µM inner reverse primer  0.5µL Phusion HF Polymerase  1ng template  Water to 50µL | 10µL 5x Phusion Buffer  1µL 10mM dNTPs  2.5µL 10µM inner forward primer  2.5µL 10µM inner reverse primer  0.5µL Phusion HF Polymerase  1ng template  Water to 50µL |
| Cycle Conditions | 98°C for 30s  25 cycles of:  98°C for 5s  53°C for 15s  72°C for 15s  72°C for 10 min | 98°C for 30s  16 cycles of:  98°C for 5s  53°C for 15s  72°C for 15s  72°C for 10 min | 98°C for 30s  9 cycles of:  98°C for 5s  53°C for 15s  72°C for 15s  72°C for 10 min |
| Added | N/A | 1.875µL 1:10 diluted ExoI | 0.5µL Phusion HF Polymerase  2.5µL 10µM outer forward primer  2.5µL 10µM outer reverse primer |
| Cycles | N/A | 37°C for 30 min  95°C for 5 min | 98°C for 30s  14 cycles of:  98°C for 5s  53°C for 15s  72°C for 15s  72°C for 10 min |
| Added | N/A | In new PCR tube:  10µL 5x Phusion Buffer  1µL 10mM dNTPs  2.5µL 10µM inner forward primer  2.5µL 10µM inner reverse primer  0.5µL Phusion HF Polymerase  1µL product from prev. step  Water to 50µL | N/A |
| Cycles | N/A | 98°C for 30s  16 cycles of:  98°C for 5s  53°C for 15s  72°C for 15s  72°C for 10 min | N/A |
