# Supplementary material for: High-Resolution Sequence-Function Mapping of Full-Length Proteins
Source: PLoS One. 2015 Mar 19;10(3):e0118193. doi: 10.1371/journal.pone.0118193 (PMC4366243; doi:10.1371/journal.pone.0118193)
Supplement: S4 Table — Results from double transformation experiments. (DOCX) [file pone.0118193.s006.docx]

**Table S4. Experimental results used for determination of double transformation percentage.**

| ***Mass of Plasmid (ng)*** | ***Plasmid Antibiotic Resistance*** | ***Number of CFU's on Plate*** | | | ***% Double Transformants*** |
| --- | --- | --- | --- | --- | --- |
|  |  | **Amp Plate** | **Kan Plate** | **Amp+Kan Plate** |  |
| 0 | - | <10 | <10 | <10 | N/A |
| 10 | Amp | 690,000 | <10 | <10 | N/A |
| 5 | Kan | <10 | 250,000 | <10 | N/A |
| 40 | 100:1 Amp:Kan | 2,200,000 | 16000 | 310 | 1.9% |
