# Supplementary material for: High-Resolution Sequence-Function Mapping of Full-Length Proteins
Source: PLoS One. 2015 Mar 19;10(3):e0118193. doi: 10.1371/journal.pone.0118193 (PMC4366243; doi:10.1371/journal.pone.0118193)
Supplement: S1 Note — (DOCX) [file pone.0118193.s007.docx]

**Note S1. Gene Sequences**

Legend

Red: proJK1 promoter

Blue: -35 Sequence

Magenta: -10 Sequence

Green: RBS

Underlined: Coding sequence for LGK

>pJK_proJK1_LGK

TGGCGAATGGGACGCGCCCTGTAGCGGCGCATTAAGCGCGGCGGGTGTGGTGGTTACGCGCAGCGTGACCGCTACACTTGCCAGCGCCCTAGCGCCCGCTCCTTTCGCTTTCTTCCCTTCCTTTCTCGCCACGTTCGCCGGCTTTCCCCGTCAAGCTCTAAATCGGGGGCTCCCTTTAGGGTTCCGATTTAGTGCTTTACGGCACCTCGACCCCAAAAAACTTGATTAGGGTGATGGTTCACGTAGTGGGCCATCGCCCTGATAGACGGTTTTTCGCCCTTTGACGTTGGAGTCCACGTTCTTTAATAGTGGACTCTTGTTCCAAACTGGAACAACACTCAACCCTATCTCGGTCTATTCTTTTGATTTATAAGGGATTTTGCCGATTTCGGCCTATTGGTTAAAAAATGAGCTGATTTAACAAAAATTTAACGCGAATTTTAACAAAATATTAACGTTTACAATTTCAGGTGGCACTTTTCGGGGAAATGTGCGCGGAACCCCTATTTGTTTATTTTTCTAAATACATTCAAATATGTATCCGCTCATGAATTAATTCTTACCAATGCTTAATCAGTGAGGCACCTATCTCAGCGATCTGTCTATTTCGTTCATCCATAGTTGCCTGACTCCCCGTCGTGTAGATAACTACGATACGGGAGGGCTTACCATCTGGCCCCAGTGCTGCAATGATACCGCGAGACCCACGCTCACCGGCTCCAGATTTATCAGCAATAAACCAGCCAGCCGGAAGGGCCGAGCGCAGAAGTGGTCCTGCAACTTTATCCGCCTCCATCCAGTCTATTAATTGTTGCCGGGAAGCTAGAGTAAGTAGTTCGCCAGTTAATAGTTTGCGCAACGTTGTTGCCATTGCTGCAGGCATCGTGGTGTCACGCTCGTCGTTTGGTATGGCTTCATTCAGCTCCGGTTCCCAACGATCAAGGCGAGTTACATGATCCCCCATGTTGTGCAAAAAAGCGGTTAGCTCCTTCGGTCCTCCGATCGTTGTCAGAAGTAAGTTGGCCGCAGTGTTATCACTCATGGTTATGGCAGCACTGCATAATTCTCTTACTGTCATGCCATCCGTAAGATGCTTTTCTGTGACTGGTGAGTACTCAACCAAGTCATTCTGAGAATAGTGTATGCGGCGACCGAGTTGCTCTTGCCCGGCGTCAATACGGGATAATACCGCGCCACATAGCAGAACTTTAAAAGTGCTCATCATTGGAAAACGTTCTTCGGGGCGAAAACTCTCAAGGATCTTACCGCTGTTGAGATCCAGTTCGATGTAACCCACTCGTGCACCCAACTGATCTTCAGCATCTTTTACTTTCACCAGCGTTTCTGGGTGAGCAAAAACAGGAAGGCAAAATGCCGCAAAAAAGGGAATAAGGGCGACACGGAAATGTTGAATACTCATAACACCCCTTGTATTACTGTTTATGTAAGCAGACAGTTTTATTGTTCATGACCAAAATCCCTTAACGTGAGTTTTCGTTCCACTGAGCGTCAGACCCCGTAGAAAAGATCAAAGGATCTTCTTGAGATCCTTTTTTTCTGCGCGTAATCTGCTGCTTGCAAACAAAAAAACCACCGCTACCAGCGGTGGTTTGTTTGCCGGATCAAGAGCTACCAACTCTTTTTCCGAAGGTAACTGGCTTCAGCAGAGCGCAGATACCAAATACTGTCCTTCTAGTGTAGCCGTAGTTAGGCCACCACTTCAAGAACTCTGTAGCACCGCCTACATACCTCGCTCTGCTAATCCTGTTACCAGTGGCTGCTGCCAGTGGCGATAAGTCGTGTCTTACCGGGTTGGACTCAAGACGATAGTTACCGGATAAGGCGCAGCGGTCGGGCTGAACGGGGGGTTCGTGCACACAGCCCAGCTTGGAGCGAACGACCTACACCGAACTGAGATACCTACAGCGTGAGCTATGAGAAAGCGCCACGCTTCCCGAAGGGAGAAAGGCGGACAGGTATCCGGTAAGCGGCAGGGTCGGAACAGGAGAGCGCACGAGGGAGCTTCCAGGGGGAAACGCCTGGTATCTTTATAGTCCTGTCGGGTTTCGCCACCTCTGACTTGAGCGTCGATTTTTGTGATGCTCGTCAGGGGGGCGGAGCCTATGGAAAAACGCCAGCAACGCGGCCTTTTTACGGTTCCTGGCCTTTTGCTGGCCTTTTGCTCACATGTTCTTTCCTGCGTTATCCCCTGATTCTGTGGATAACCGTATTACCGCCTTTGAGTGAGCTGATACCGCTCGCCGCAGCCGAACGACCGAGCGCAGCGAGTCAGTGAGCGAGGAAGCGGAAGAGCGCCTGATGCGGTATTTTCTCCTTACGCATCTGTGCGGTATTTCACACCGCATATATGGTGCACTCTCAGTACAATCTGCTCTGATGCCGCATAGTTAAGCCAGTATACACTCCGCTATCGCTACGTGACTGGGTCATGGCTGCGCCCCGACACCCGCCAACACCCGCTGACGCGCCCTGACGGGCTTGTCTGCTCCCGGCATCCGCTTACAGACAAGCTGTGACCGTCTCCGGGAGCTGCATGTGTCAGAGGTTTTCACCGTCATCACCGAAACGCGCGAGGCAGCTGCGGTAAAGCTCATCAGCGTGGTCGTGAAGCGATTCACAGATGTCTGCCTGTTCATCCGCGTCCAGCTCGTTGAGTTTCTCCAGAAGCGTTAATGTCTGGCTTCTGATAAAGCGGGCCATGTTAAGGGCGGTTTTTTCCTGTTTGGTCACTGATGCCTCCGTGTAAGGGGGATTTCTGTTCATGGGGGTAATGATACCGATGAAACGAGAGAGGATGCTCACGATACGGGTTACTGATGATGAACATGCCCGGTTACTGGAACGTTGTGAGGGTAAACAACTGGCGGTATGGATGCGGCGGGACCAGAGAAAAATCACTCAGGGTCAATGCCAGCGCTTCGTTAATACAGATGTAGGTGTTCCACAGGGTAGCCAGCAGCATCCTGCGATGCAGATCCGGAACATAATGGTGCAGGGCGCTGACTTCCGCGTTTCCAGACTTTACGAAACACGGAAACCGAAGACCATTCATGTTGTTGCTCAGGTCGCAGACGTTTTGCAGCAGCAGTCGCTTCACGTTCGCTCGCGTATCGGTGATTCATTCTGCTAACCAGTAAGGCAACCCCGCCAGCCTAGCCGGGTCCTCAACGACAGGAGCACGATCATGCGCACCCGTGGGGCCGCCATGCCGGCCACAGCTAACACCACGTCGTCCCTATCTGCTGCCCTAGGTCTATGAGTGGTTGCTGGATAACTTTATGGGCATGCATAAGGCTCGTATAATATATTCAGGGAGACCACAACGGTTTCCCTCTACAAATAATTTTGTTTAACTTTCTAGAAATAATTTTGTTTAACTTTAAGAAGGAGATATACATATGCCGATTGCGACCTCAACGGGTGATAATGTTCTGGACTTTACGGTTCTGGGCCTGAATAGCGGTACGAGTATGGATGGTATTGACTGCGCACTGTGTCATTTCTATCAGAAAACCCCGGATGCTCCGATGGAATTTGAACTGCTGGAATACGGCGAAGTTCCGCTGGCGCAGCCGATTAAACAACGTGTCATGCGCATGATCCTGGAAGATACCACGAGCCCGTCTGAACTGTCAGAAGTCAACGTGATTCTGGGTGAACATTTTGCGGATGCCGTCCGTCAGTTCGCGGCCGAACGCAATGTGGATCTGTCAACCATTGACGCAATCGCTTCGCACGGCCAGACGATTTGGCTGCTGAGTATGCCGGAAGAAGGTCAAGTGAAATCCGCCCTGACCATGGCAGAAGGCGCTATCCTGGCGAGTCGTACGGGTATTACCTCCATCACGGATTTCCGTATTTCCGACCAGGCAGCTGGTCGTCAAGGTGCACCGCTGATCGCATTTTTCGATGCTCTGCTGCTGCATCACCCGACCAAACTGCGCGCGTGCCAGAACATTGGCGGTATCGCCAATGTGTGTTTTATTCCGCCGGATGTTGACGGCCGTCGCACCGATGAATATTACGATTTTGACACGGGTCCGGGCAACGTGTTCATCGACGCAGTGGTTCGTCATTTTACCAATGGTGAACAGGAATATGATAAAGACGGTGCTATGGGCAAACGCGGTAAAGTCGATCAGGAACTGGTGGATGACTTTCTGAAAATGCCGTATTTCCAACTGGACCCGCCGAAAACCACGGGCCGTGAAGTTTTTCGCGATACCCTGGCACATGACCTGATTCGTCGCGCGGAAGCCAAAGGTCTGAGCCCGGATGACATCGTGGCCACCACGACCCGTATTACGGCACAGGCTATCGTTGATCACTATCGTCGCTACGCGCCGTCACAAGAAATTGACGAAATCTTCATGTGCGGCGGTGGCGCCTATAACCCGAATATTGTGGAATTTATCCAGCAATCGTACCCGAACACCAAAATTATGATGCTGGATGAAGCAGGTGTCCCGGCAGGTGCAAAAGAAGCGATTACGTTCGCCTGGCAGGGCATGGAAGCCCTGGTTGGTCGTAGCATCCCGGTTCCGACCCGTGTCGAAACGCGCCAGCACTATGTGCTGGGCAAAGTTAGCCCGGGTCTGAATTACCGCTCTGTGATGAAAAAAGGCATGGCATTTGGTGGCGATGCTCAGCAACTGCCGTGGGTTTCTGAAATGATCGTGAAGAAAAAAGGCAAAGTTATCACCAACAACTGGGCGCTCGAGCACCACCACCACCACCACTGAGATCCGGCTGCTAACAAAGCCCGAAAGGAAGCTGAGTTGGCTGCTGCCACCGCTGAGCAATAACTAGCATAACCCCTTGGGGCCTCTAAACGGGTCTTGAGGGGTTTTTTGCTGAAAGGAGGAACTATATCCGGAT

>pJK_proJK1_kanR_LGK

TGGCGAATGGGACGCGCCCTGTAGCGGCGCATTAAGCGCGGCGGGTGTGGTGGTTACGCGCAGCGTGACCGCTACACTTGCCAGCGCCCTAGCGCCCGCTCCTTTCGCTTTCTTCCCTTCCTTTCTCGCCACGTTCGCCGGCTTTCCCCGTCAAGCTCTAAATCGGGGGCTCCCTTTAGGGTTCCGATTTAGTGCTTTACGGCACCTCGACCCCAAAAAACTTGATTAGGGTGATGGTTCACGTAGTGGGCCATCGCCCTGATAGACGGTTTTTCGCCCTTTGACGTTGGAGTCCACGTTCTTTAATAGTGGACTCTTGTTCCAAACTGGAACAACACTCAACCCTATCTCGGTCTATTCTTTTGATTTATAAGGGATTTTGCCGATTTCGGCCTATTGGTTAAAAAATGAGCTGATTTAACAAAAATTTAACGCGAATTTTAACAAAATATTAACGTTTACAATTTCAGGTGGCACTTTTCGGGGAAATGTGCGCGGAACCCCTATTTGTTTATTTTTCTAAATACATTCAAATATGTATCCGCTCATGAATTAATTCTTAGAAAAACTCATCGAGCATCAAATGAAACTGCAATTTATTCATATCAGGATTATCAATACCATATTTTTGAAAAAGCCGTTTCTGTAATGAAGGAGAAAACTCACCGAGGCAGTTCCATAGGATGGCAAGATCCTGGTATCGGTCTGCGATTCCGACTCGTCCAACATCAATACAACCTATTAATTTCCCCTCGTCAAAAATAAGGTTATCAAGTGAGAAATCACCATGAGTGACGACTGAATCCGGTGAGAATGGCAAAAGTTTATGCATTTCTTTCCAGACTTGTTCAACAGGCCAGCCATTACGCTCGTCATCAAAATCACTCGCATCAACCAAACCGTTATTCATTCGTGATTGCGCCTGAGCGAGACGAAATACGCGATCGCTGTTAAAAGGACAATTACAAACAGGAATCGAATGCAACCGGCGCAGGAACACTGCCAGCGCATCAACAATATTTTCACCTGAATCAGGATATTCTTCTAATACCTGGAATGCTGTTTTCCCGGGGATCGCAGTGGTGAGTAACCATGCATCATCAGGAGTACGGATAAAATGCTTGATGGTCGGAAGAGGCATAAATTCCGTCAGCCAGTTTAGTCTGACCATCTCATCTGTAACATCATTGGCAACGCTACCTTTGCCATGTTTCAGAAACAACTCTGGCGCATCGGGCTTCCCATACAATCGATAGATTGTCGCACCTGATTGCCCGACATTATCGCGAGCCCATTTATACCCATATAAATCAGCATCCATGTTGGAATTTAATCGCGGCCTAGAGCAAGACGTTTCCCGTTGAATATGGCTCATAACACCCCTTGTATTACTGTTTATGTAAGCAGACAGTTTTATTGTTCATGACCAAAATCCCTTAACGTGAGTTTTCGTTCCACTGAGCGTCAGACCCCGTAGAAAAGATCAAAGGATCTTCTTGAGATCCTTTTTTTCTGCGCGTAATCTGCTGCTTGCAAACAAAAAAACCACCGCTACCAGCGGTGGTTTGTTTGCCGGATCAAGAGCTACCAACTCTTTTTCCGAAGGTAACTGGCTTCAGCAGAGCGCAGATACCAAATACTGTCCTTCTAGTGTAGCCGTAGTTAGGCCACCACTTCAAGAACTCTGTAGCACCGCCTACATACCTCGCTCTGCTAATCCTGTTACCAGTGGCTGCTGCCAGTGGCGATAAGTCGTGTCTTACCGGGTTGGACTCAAGACGATAGTTACCGGATAAGGCGCAGCGGTCGGGCTGAACGGGGGGTTCGTGCACACAGCCCAGCTTGGAGCGAACGACCTACACCGAACTGAGATACCTACAGCGTGAGCTATGAGAAAGCGCCACGCTTCCCGAAGGGAGAAAGGCGGACAGGTATCCGGTAAGCGGCAGGGTCGGAACAGGAGAGCGCACGAGGGAGCTTCCAGGGGGAAACGCCTGGTATCTTTATAGTCCTGTCGGGTTTCGCCACCTCTGACTTGAGCGTCGATTTTTGTGATGCTCGTCAGGGGGGCGGAGCCTATGGAAAAACGCCAGCAACGCGGCCTTTTTACGGTTCCTGGCCTTTTGCTGGCCTTTTGCTCACATGTTCTTTCCTGCGTTATCCCCTGATTCTGTGGATAACCGTATTACCGCCTTTGAGTGAGCTGATACCGCTCGCCGCAGCCGAACGACCGAGCGCAGCGAGTCAGTGAGCGAGGAAGCGGAAGAGCGCCTGATGCGGTATTTTCTCCTTACGCATCTGTGCGGTATTTCACACCGCATATATGGTGCACTCTCAGTACAATCTGCTCTGATGCCGCATAGTTAAGCCAGTATACACTCCGCTATCGCTACGTGACTGGGTCATGGCTGCGCCCCGACACCCGCCAACACCCGCTGACGCGCCCTGACGGGCTTGTCTGCTCCCGGCATCCGCTTACAGACAAGCTGTGACCGTCTCCGGGAGCTGCATGTGTCAGAGGTTTTCACCGTCATCACCGAAACGCGCGAGGCAGCTGCGGTAAAGCTCATCAGCGTGGTCGTGAAGCGATTCACAGATGTCTGCCTGTTCATCCGCGTCCAGCTCGTTGAGTTTCTCCAGAAGCGTTAATGTCTGGCTTCTGATAAAGCGGGCCATGTTAAGGGCGGTTTTTTCCTGTTTGGTCACTGATGCCTCCGTGTAAGGGGGATTTCTGTTCATGGGGGTAATGATACCGATGAAACGAGAGAGGATGCTCACGATACGGGTTACTGATGATGAACATGCCCGGTTACTGGAACGTTGTGAGGGTAAACAACTGGCGGTATGGATGCGGCGGGACCAGAGAAAAATCACTCAGGGTCAATGCCAGCGCTTCGTTAATACAGATGTAGGTGTTCCACAGGGTAGCCAGCAGCATCCTGCGATGCAGATCCGGAACATAATGGTGCAGGGCGCTGACTTCCGCGTTTCCAGACTTTACGAAACACGGAAACCGAAGACCATTCATGTTGTTGCTCAGGTCGCAGACGTTTTGCAGCAGCAGTCGCTTCACGTTCGCTCGCGTATCGGTGATTCATTCTGCTAACCAGTAAGGCAACCCCGCCAGCCTAGCCGGGTCCTCAACGACAGGAGCACGATCATGCGCACCCGTGGGGCCGCCATGCCGGCCACAGCTAACACCACGTCGTCCCTATCTGCTGCCCTAGGTCTATGAGTGGTTGCTGGATAACTTTATGGGCATGCATAAGGCTCGTATAATATATTCAGGGAGACCACAACGGTTTCCCTCTACAAATAATTTTGTTTAACTTTCTAGAAATAATTTTGTTTAACTTTAAGAAGGAGATATACATATGCCGATTGCGACCTCAACGGGTGATAATGTTCTGGACTTTACGGTTCTGGGCCTGAATAGCGGTACGAGTATGGATGGTATTGACTGCGCACTGTGTCATTTCTATCAGAAAACCCCGGATGCTCCGATGGAATTTGAACTGCTGGAATACGGCGAAGTTCCGCTGGCGCAGCCGATTAAACAACGTGTCATGCGCATGATCCTGGAAGATACCACGAGCCCGTCTGAACTGTCAGAAGTCAACGTGATTCTGGGTGAACATTTTGCGGATGCCGTCCGTCAGTTCGCGGCCGAACGCAATGTGGATCTGTCAACCATTGACGCAATCGCTTCGCACGGCCAGACGATTTGGCTGCTGAGTATGCCGGAAGAAGGTCAAGTGAAATCCGCCCTGACCATGGCAGAAGGCGCTATCCTGGCGAGTCGTACGGGTATTACCTCCATCACGGATTTCCGTATTTCCGACCAGGCAGCTGGTCGTCAAGGTGCACCGCTGATCGCATTTTTCGATGCTCTGCTGCTGCATCACCCGACCAAACTGCGCGCGTGCCAGAACATTGGCGGTATCGCCAATGTGTGTTTTATTCCGCCGGATGTTGACGGCCGTCGCACCGATGAATATTACGATTTTGACACGGGTCCGGGCAACGTGTTCATCGACGCAGTGGTTCGTCATTTTACCAATGGTGAACAGGAATATGATAAAGACGGTGCTATGGGCAAACGCGGTAAAGTCGATCAGGAACTGGTGGATGACTTTCTGAAAATGCCGTATTTCCAACTGGACCCGCCGAAAACCACGGGCCGTGAAGTTTTTCGCGATACCCTGGCACATGACCTGATTCGTCGCGCGGAAGCCAAAGGTCTGAGCCCGGATGACATCGTGGCCACCACGACCCGTATTACGGCACAGGCTATCGTTGATCACTATCGTCGCTACGCGCCGTCACAAGAAATTGACGAAATCTTCATGTGCGGCGGTGGCGCCTATAACCCGAATATTGTGGAATTTATCCAGCAATCGTACCCGAACACCAAAATTATGATGCTGGATGAAGCAGGTGTCCCGGCAGGTGCAAAAGAAGCGATTACGTTCGCCTGGCAGGGCATGGAAGCCCTGGTTGGTCGTAGCATCCCGGTTCCGACCCGTGTCGAAACGCGCCAGCACTATGTGCTGGGCAAAGTTAGCCCGGGTCTGAATTACCGCTCTGTGATGAAAAAAGGCATGGCATTTGGTGGCGATGCTCAGCAACTGCCGTGGGTTTCTGAAATGATCGTGAAGAAAAAAGGCAAAGTTATCACCAACAACTGGGCGCTCGAGCACCACCACCACCACCACTGAGATCCGGCTGCTAACAAAGCCCGAAAGGAAGCTGAGTTGGCTGCTGCCACCGCTGAGCAATAACTAGCATAACCCCTTGGGGCCTCTAAACGGGTCTTGAGGGGTTTTTTGCTGAAAGGAGGAACTATATCCGGAT

> levoglucosan kinase [Lipomyces starkeyi] | (49437.16 Da)

MPIATSTGDNVLDFTVLGLNSGTSMDGIDCALCHFYQKTPDAPMEFELLEYGEVPLAQPI

KQRVMRMILEDTTSPSELSEVNVILGEHFADAVRQFAAERNVDLSTIDAIASHGQTIWLL

SMPEEGQVKSALTMAEGAILASRTGITSITDFRISDQAAGRQGAPLIAFFDALLLHHPTK

LRACQNIGGIANVCFIPPDVDGRRTDEYYDFDTGPGNVFIDAVVRHFTNGEQEYDKDGAMGKRGKVDQELVDDFLKMPYFQLDPPKTTGREVFRDTLAHDLIRRAEAKGLSPDDIVATTTRITAQAIVDHYRRYAPSQEIDEIFMCGGGAYNPNIVEFIQQSYPNTKIMMLDEAGVPAGAKEAITFAWQGMEALVGRSIPVPTRVETRQHYVLGKVSPGLNYRSVMKKGMAFGGDAQQLPWVSEMIVKKKGKVITNNWALEHHHHHH
