# Supplementary material for: High-Resolution Sequence-Function Mapping of Full-Length Proteins
Source: PLoS One. 2015 Mar 19;10(3):e0118193. doi: 10.1371/journal.pone.0118193 (PMC4366243; doi:10.1371/journal.pone.0118193)
Supplement: S2 Note — (DOCX) [file pone.0118193.s008.docx]

**Note S2: Practical Considerations**

**Target Selection**

*INPUT: Target protein*

*OUTPUT: Selection conditions, Protein gene sequence*

At this step the most important consideration is that, for a given protein target, there are conditions under which more active variants can be selected over less active variants. In this paper we explicitly consider growth-based selections and fluorescence activated cell sorting (FACS) screening techniques.

For growth-based selections, a common strategy is to identify conditions where growth depends on flux through a given pathway. For example, bacterial growth in the presence of beta-lactam antibiotics requires a certain enzymatic flux of beta-lactamase. To allow determination of more active proteins than the starting sequence, conditions should be screened where the starting sequence does not support maximal growth in the host background. On the other hand, the growth rate should be significant enough that the selection experiment can be completed in 1-2 days.

FACS, in conjunction with cell display techniques like yeast-surface display, can be used to evaluate protein-protein binders, protein-small molecule binders, and even certain enzymes. In all cases, higher fluorescence (after labeling) is correlated with more potent activity. Conditions should be screened such that the starting sequence does not support maximum fluorescence. In the case of protein binders, one key parameter to vary is the labeling concentration relative to the dissociation constant of the interaction for the starting sequence. At labeling concentrations well above the dissociation constant, increases in binding affinity result in minimal fluorescence increases. The set up and validation of the selection system is key to the efficiency and results of the experiment. A poorly set up selection will yield poor sequence-function maps.

### **Gene Tiling**

*INPUT: Protein gene sequence, sequencing read length*

*OUTPUT: Gene tiling scheme, “inner” primer sequences*

The gene sequence needs to be segmented into tiles for library preparation and selections to allow efficient mutation counting with Illumina sequencing reads. Genes are tiled in segments of lengths that are multiples of three and at least 20 base pairs shorter than the sequencing reads. Tiles are no longer than 126 bp for 150 bp paired-end (150bp PE) reads. Each section should be at a length of a multiple of three (i.e., 117, 120, 123, etc.) as to not split an amino acid codon over multiple sections. We have written a custom script (**Script S2;** GeneTiles.m**)** to facilitate tiling and design of the inner primers. The input into the program is the gene sequence (including 33 bp up and downstream of the gene sequence) and the read length for the next generation sequencing. The output from this program is spatial location of individual gene tiles, and the set of inner primers needed for sequencing preparation.

To use GeneTile.m:

1. Create a file named “genesequence.txt” in FASTA format with your gene sequence. Include exactly 33 bp upstream and downstream of your genesequence in this file.
2. Load “GeneTile.m” and “genesequence.txt” into MATLAB and run the function GeneTile.m
3. The output file containing the spatial location of the gene tiles and the inner primer sequences.

**Library Preparation**

*INPUT: Pfunkel Method, QuikChange Primer Sequences, Gene tiling scheme*

*OUTPUT: Libraries for selection*

SSM libraries should be prepared using the Pfunkel Method essentially as described [1]. As stated in the results section, we recommend using the Agilent QuikChange Primer Designer (http://www.genomics.agilent.com/primerDesignProgram.jsp?_DARGS=/primerDesignProgram.jsp). Although it is much more labor intensive up-front, in our hands the longer lengths of the primers allow better library coverage than the primer design script provided by Firnberg *et al.* [1]. It is important to prepare the library of each tile separately, as each tile needs to be selected independently. Library coverage statistics can be calculated following Boder and Wittrup [2]; the number of transformants from each tiled library should exceed the theoretical library size by at least 7-fold (>99.9% coverage). The library for each tile is plated onto a separate Bioassay plate, and then plasmid DNA is recovered by Qiagen Midiprep.

This library plasmid DNA can then be transformed into the strain used for selection. In the specific case of *S. cerevisiae*, high efficiency transformations can be done following Benatuil et.al. [3]. The high number of transformants obtained from this method almost always supports the degeneracy of SSM libraries. For both *S. cerevisiae* and *E. coli*- based libraries, cell stocks can be stored at -80°C until used for selections.

Double transformation artifacts do not need to be considered if transforming a low copy number plasmid (like in yeast surface display). Double transformation artifacts do need to be considered when transforming with medium or high copy number plasmids. Under the model developed in this paper, no correction needs to be made for growth-based selections using 10 or less average population doublings at double transformation percentages less than 10%. Our recommendation is that for growth-based selections you vary the transformation parameters (cell density, amount of plasmid DNA, etc.) until the transformation efficiency can support the degeneracy of the library and the percentage of double transformants is less than 10%.

**Selections**

*INPUT: Libraries, Selection Conditions*

*OUTPUT: Selected Libraries*

Selections should be performed following the experimental conditions explained in the **Results** section. We recommend growth-based selections be run for 6-8 average population doublings. Growth-based selections should be performed on exponentially growing cells only (avoid lag phases and post-exponential growth phases). The initial inoculum density should be much larger than the number of variants in the library population. Following selection, cells can be stored in glycerol stocks at -80^o^C before library DNA is prepared for deep sequencing. For FACS, collecting the top 5% of the population using a square gate (one color sorting). To generate good counting statistics, at least 100x of the theoretical library size should be collected. For a library size of 2500, this means 250,000 cells collected (5,000,000 cells sorted). In the specific case of yeast-surface display, cells should be labeled with biotinylated antigen at 50% of the dissociation constant for the wild-type interaction. Following sorting, yeast should be recovered in selective media and stored in 20 mM HEPES 150 mM NaCl pH 7.5, 20% (w/v) glycerol in 1x10^7^ aliquots at −80 °C until they are prepared for deep sequencing.

**Deep Sequencing Preparation**

*INPUT: Selected Libraries, Unselected Libraries, Inner/Outer Primers*

*OUTPUT: Libraries ready for next generation sequencing*

For next-generation sequencing we utilized the Illumina MiSeq 150-bp PE reads. This technology has recently been expanded to 300 bp pe reads, as using longer reads reduces the number of libraries and selections. Inner primers are designed using the gene tiling script (**Script S2)**. The inner primers follow the pattern:

FWD: 5’- GTTCAGAGTTCTACAGTCCGACGATC<SEGMENT OVERLAP>-3’

REV: 5’- CCTTGGCACCCGAGAATTCCA<SEGEMENT OVERLAP REV. COMP.>-3’

Outer primers are taken from the Illumina TrueSeq Small RNA kit and can be ordered using the sequences listed in **Table S2.** The outer primers are meant to be universal: while the same forward primer is used for each library member, the reverse primers contain barcodes for multiplexing. Each library (sorted and unsorted) should use its own barcode to allow demultiplexing of sequencing reads. To append the primers, DNA is first extracted from the stored libraries. Plasmid DNA can be extracted from *E. coli* using a Qiagen miniprep, while for *S. cerevisiae* we use by a modified smash and grab protocol (**Note S4).** Primers are attached to the gene tile using PCR method A found in **Table S3.** PCR reactions are purified using AMPure beads and quantified using Quant-it PicoGreen dsDNA Assay on a plate reader. Libraries are mixed in equimolar amounts to ensure even sequencing before being delivered for sequencing.

**Data Analysis**

*INPUT: Sequencing reads*

*OUTPUT: Normalized fitness metrics for the sequence-function landscape*

A modified Enrich-0.2 program is used to interpret and analyze the sequencing data [4]. Modifications to the Enrich-0.2 program were made according to **Script S1**. Currently Enrich-0.2 will only translate proteins from the first base pair of the dna sequence. Using the gene-tiling method the protein sequence is not always in the same frame as the first base pair. The modifications to Enrich-0.2 allows for this capability. Using a new <TRANSLATE_START> command into the config file where you indicate the beginning of translation of the DNA to the protein sequence. We also found it necessary to edit the length of the initial matching protein sequence to 10 bp over the standard 20 found in the Enrich program since the flanking sequences to the tiles are not usually longer than 20 base pairs. The script for the enrich patch is called EnrichPatch.py to use the patch, move it into the main enrich directory and use the command line “python EnrichPatch.py” this should modify Enrich for your purposes. To ensure proper execution of the patch, the ‘example_local_config’ file should contain a new <translate_start> command line in the read_aligner section.

Enrich outputs “unlink_wild_counts” for both the selected and unselected populations, at both the dna and protein levels. These files are populated frequency matrices the rows being residue position, relative to the beginning of translation for that section, and the columns are the different amino acid residues. The frequency of each variant in the libraries are listed. These files can be used to determine the log_2_ enrichment ratio for each variant. Apart from the frequency matrices it is important to extract the wild-type enrichment ratio for each gene tile, from the “ratios_sel_PRO” file for the NA_NA variant to use in the normalization equations. The normalization equations, for the fitness metric, that should be use are:

 (11)

For growth-based selections and,

 (20)

for FACS selections. Note the need for the enrichment ratio of both the variant *i* and the wild-type along with the number of population doubling times for the growth-based selections and the log transformed standard deviation for the FACS based selections. We use custom scripts to do the normalization of the data and import the normalized fitness metrics into Excel in order to visualize the fitness landscapes using the conditional formatting options.

**Supporting References**

1. Firnberg E, Ostermeier M (2012) PFunkel: efficient, expansive, user-defined mutagenesis. PLoS One 7: e52031.

2. Boder ET, Wittrup KD (1998) Optimal Screen of Surface-Displayed Polypeptide Libraries BIotechnolgical Progress 1998: 55-62.

3. Benatuil L, Perez JM, Belk J, Hsieh CM (2010) An improved yeast transformation method for the generation of very large human antibody libraries. Protein Eng Des Sel 23: 155-159.

4. Fowler DM, Araya CL, Gerard W, Fields S (2011) Enrich: software for analysis of protein function by enrichment and depletion of variants. Bioinformatics 27: 3430-3431.
