# Supplementary material for: High-Resolution Sequence-Function Mapping of Full-Length Proteins
Source: PLoS One. 2015 Mar 19;10(3):e0118193. doi: 10.1371/journal.pone.0118193 (PMC4366243; doi:10.1371/journal.pone.0118193)
Supplement: S3 Note — (DOCX) [file pone.0118193.s009.docx]

**Note S3. Effect of double transformation on enrichment ratios for growth-based selections**

Consider a microorganism transformed with a plasmid variant *i*. When grown exponentially, the time-dependent concentration of the culture (*x_fi_*) can be written:

 (S1)

where *x_oi_* is the initial concentration, *μ_i_* is the specific growth rate, and *t* is time.

Consider now a microorganism harboring two different plasmids: plasmid variant *i* and an unrelated plasmid variant *j*. For all variants 1 ≤ *j* ≤n in the population, the growth of microbes transformed with plasmid variant i and all other variants in the population (*x_fΦi_*) can be represented by:

 (S2)

Where *f_j_* represents the frequency of plasmid *j* in the sequenced population. The form of this growth equation is based on an assumption that a microbe double transformed with plasmids *i* and *j* will grow at an average of their individual growth rates (see below for further discussion).

Taking the doubly transformed population into account, the time-dependent concentration of cells harboring plasmid *i* can be written as:

 (S3)

where *ϕ* is the fraction of the population that is doubly transformed.

The actual, measured enrichment ratio can be represented by:

 (S4)

and the true enrichment ratio, defined as the enrichment ratio obtained in the absence of double transformants, can be represented by:

 (S5)

The measured enrichment ratio can also written as:

 (S6)

Where represents a correction factor to the true enrichment ratio because of the doubly transformed populations. We can further represent as:

 (S7)

Substituting (S1) and (S2) into (S7):

 (S8)

Since

 (S9)

We can rewrite (S8) as:

 (S10)

As before, time can be represented in terms of population doubling periods, $g_{p}$, and the average growth rate of the population ():

 (S11)

Combining terms, we derive the following form for the correction factor:

 (S12)

This correction factor is a function of the double transformation rate, the number of doubling periods, the average growth rate of the population, and the distribution of the starting growth rates in the beginning population. Because we do not know the distribution of growth rates in a given population *a priori*, this correction factor cannot reliably be used. However, it can help illuminate conditions where the correction factor is expected to small.

The main assumption in deriving this correction factor is that doubly transformed cells grow at an average rate of cells transformed with the individual plasmids. This assumption is likely to be correct for plasmids that are segregated evenly upon cell division, as well as where activity of an individual protein is linearly proportional to the growth rate of cells harboring its plasmid. More complicated growth models can be assessed using the framework laid out here.
