# Supplementary material for: High-Resolution Sequence-Function Mapping of Full-Length Proteins
Source: PLoS One. 2015 Mar 19;10(3):e0118193. doi: 10.1371/journal.pone.0118193 (PMC4366243; doi:10.1371/journal.pone.0118193)
Supplement: S4 Note — (DOCX) [file pone.0118193.s010.docx]

# Note S4. Protocol for Yeast Plasmid Extraction and Deep Sequencing Preparation

**The original method is from Aaron Chevalier and Tim Whitehead (01/2011)**

**Modified by Caitlin Kowalsky and Tim Whitehead (09/2014)**

**Procedure Overview:** This prep produces about ~100ng template per 10^7^ yeast cells with a low copy number plasmid. Briefly, 10^7^ yeast cells are stored as pellets in -80^o^C freezer until prep. Cells are shed of their cell wall by zymolase, and opened by a freeze-thaw cycle followed by an alkaline lysis miniprep procedure. Sheared genomic DNA and ssDNA are partially cleaned up from the plasmid DNA by an exonuclease processing step, and then the insert is amplified by PCR using a high-fidelity polymerase. PCR products are verified by agarose gel, cleaned by an ampure procedure, and quantified by Quant-it. Portions of this procedure come from Andy Sherenburgs**^1^** and Maitreya Dunham’s lab**^2^**, and are marked when appropriate. The original yeast display plasmid (pCTCON) uses a Cen6 ori, which quanitification shows gives 1-3 copies per cell.

**A) Yeast Prep^1^**

Uses Zymo Research Yeast Plasmid Miniprep II kit (Cat no: D2004) and Qiagen mini-prep.

1. Pellet 4e7 yeast cells.
2. Resuspend in 200μl Solution 1.
3. Add 5μl Zymolyase (5U/μl).
4. Incubate at 37˚C for 4hrs, mix once per hour.
5. Perform 1 freeze-thaw cycle in dry ice/EtOH bath and 42˚C.
6. Add 200μl Solution 2, mix and well and let sit 3-5min.
7. Add 400ul Solution 3, mix well and centrifuge max RPM for 5min.
8. Transfer supernatant to a Qiagen mini-prep column.
9. Spin 1 min max RPM.
10. Add 700μl PB buffer and spin 30sec at max RPM.
11. Add 700μl PE buffer and spin 30sec at max RPM.
12. Repeat PE wash.
13. Pour out supernatant and spin at max RPM for 1min to dry column.
14. Add 30μl EB to elute pin 1min max RPM.
15. Reload column with eluate and spin again.
16. Store 15μl and proceed with remaining 15μl.

The yeast prep after this step contains a large amount of sheared genomic DNA that is co-purified along with plasmid DNA. Because of subsequent PCR inhibition, this sheared genomic DNA needs to be removed before large-scale amplification.

(**OPTIONAL:** 1μl in a 50μl PCR can be amplified up (25X cycles) and run on a 2% agarose SYBRgold gel as size validation. More than 1μl in PCR will cause inhibition. SYBRgold is used as a dye because it is ca. 25X more sensitive than ethidium bromide.)

**B) Purification of plasmid from yeast prep^2^**

ExoI exonuclease I (NEB cat no: M0293S) - Catalyzes the removal of nucleotides from single-stranded DNA in the 3' to 5' direction.

Lamda exonuclease (NEB cat no: M0262S) – cleaves mononucleotides from duplex DNA 5’ to 3’.

1. Combine the following in order:

Lambda buffer 10x 2μl

DNA(from A) 15μl

Lambda 1μl

ExoI 2μl

Final volume 20μl

2. On a thermocycler, incubate 1hr 30min at 30˚C.

3. Inactivate for 20min at 80˚C.

4. Use the standard procedure for Qiagen PCR cleanup and elute in 30 μL EB.

5. After this step there is not enough DNA to see using nanodrop UV quantification. Could use qPCR or more sensitive dyes to verify concentration.

**6.**  Store 15 μL and proceed with remaining 15 μL.

**D) Large Scale Amplification and Appending of Illumina Adaptor Sequences**

Use Method A as indicated in the manuscript.

**E) Clean up with Agencourt AMPure XP**

1. Use gel electrophoresis to validate that the PCR product is of the correct size. Use a small portion of the reaction (5-10 μL only).

2. Follow the standard protocol on the remainder of the PCR reaction.

**F) Quantification using Quant-it PicoGreen dsDNA Assay Kit**

Follow the standard procedure for Quant-it to quantify the PCR product. The final yield should be about 1-4 ng/μl in 40 μl.
